# Identification of transcripts with enriched expression in the developing and adult pancreas

**DOI:** 10.1186/gb-2008-9-6-r99

**Published:** 2008-06-14

**Authors:** Brad G Hoffman, Bogard Zavaglia, Joy Witzsche, Teresa Ruiz de Algara, Mike Beach, Pamela A Hoodless, Steven JM Jones, Marco A Marra, Cheryl D Helgason

**Affiliations:** 1 Department of Cancer Endocrinology, BC Cancer Research Center, West 10th Ave, Vancouver, BC, V5Z 1L3, Canada; 2 Terry Fox Laboratory, BC Cancer Research Center, West 10th Ave, Vancouver, BC, V5Z 1L3, Canada; 3 Department of Medical Genetics, Faculty of Medicine, University of British Columbia, University Boulevard, Vancouver, BC, V6T 1Z3, Canada; 4 Micheal Smith Genome Sciences Centre, BC Cancer Agency, West 7th Ave, Vancouver, BC, V5Z 4S6, Canada; 5 Department of Surgery, Faculty of Medicine, University of British Columbia, West 10th Avenue, Vancouver, BC, V5Z 4E3, Canada

## Abstract

**Background:**

Despite recent advances, the transcriptional hierarchy driving pancreas organogenesis remains largely unknown, in part due to the paucity of comprehensive analyses. To address this deficit we generated ten SAGE libraries from the developing murine pancreas spanning Theiler stages 17-26, making use of available *Pdx1 *enhanced green fluorescent protein (EGFP) and *Neurog3 *EGFP reporter strains, as well as tissue from adult islets and ducts.

**Results:**

We used a specificity metric to identify 2,536 tags with pancreas-enriched expression compared to 195 other mouse SAGE libraries. We subsequently grouped co-expressed transcripts with differential expression during pancreas development using *K*-means clustering. We validated the clusters first using quantitative real time PCR and then by analyzing the Theiler stage 22 pancreas *in situ *hybridization staining patterns of over 600 of the identified genes using the *GenePaint *database. These were then categorized into one of the five expression domains within the developing pancreas. Based on these results we identified a cascade of transcriptional regulators expressed in the endocrine pancreas lineage and, from this, we developed a predictive regulatory network describing beta-cell development.

**Conclusion:**

Taken together, this work provides evidence that the SAGE libraries generated here are a valuable resource for continuing to elucidate the molecular mechanisms regulating pancreas development. Furthermore, our studies provide a comprehensive analysis of pancreas development, and insights into the regulatory networks driving this process are revealed.

## Abstract 2

The expression profile of different developmental stages of the murine pancreas and predictions of transcription factor interactions, provides a framework for pancreas regulatory networks and development.

## Background

An understanding of the molecular and cellular regulation of pancreas development is emerging [1-5]. Expression of the transcription factor *Pdx1 *is essential for pancreas development and is initiated at Theiler stage (TS) 13 in the region of gut endoderm destined to become the pancreas [6-8]. At TS14, the foregut endoderm evaginates to form the dorsal pancreas bud [6,9,10]. The ventral bud appears somewhat later (TS17-TS20). Expression of *Ptf1a*, another critical regulatory factor, is detected at this stage and is essential for the generation of both exocrine and endocrine cell types [11-13]. The 'secondary transition', from TS20 to TS22, marks the differentiation of pancreas precursors into endocrine and exocrine cell types. The notch signaling pathway plays a critical role in this process through the lateral inhibition of neighboring cells [2,3,14,15]. Subsequently, endocrine progenitors express the essential basic helix-loop-helix transcription factor *Neurog3 *[16-18]. In response to *Neurog3 *expression, endocrine precursor cells express a number of transcriptional regulators, including *B2*/*NeuroD*, *Pax6*, *Isl1*, *Nkx2-2*, *Nkx6-1*, and others, that play roles in the differentiation and maturation of the various endocrine cells types [8,19]. By TS24 the majority of cell fates are established and remodeling of the pancreas begins with initially scattered endocrine cells formed at duct tips starting to migrate. At TS26, isletogenesis occurs as endocrine cells fuse and form recognizable 'islets', while acinar cells gain their mature ultrastructure. Pancreas development continues postnatally, with β-cells gaining the ability to sense glucose levels and respond with pulsatile insulin release.

Analysis of the transcriptomes of precursor cells present at different stages of pancreas development is expected to further facilitate a definition of the genetic cascades essential for endocrine and exocrine differentiation. Towards this end a number of microarray expression profiling studies have been carried out on the developing pancreas [20-26]. Serial analysis of gene expression (SAGE), like microarrays, provides a quantitative analysis of gene expression profiles. A major advantage of SAGE, however, is that the data are digital, making it easily shared amongst investigators and compared across different experiments and tissues.

In this study we describe the construction and analyses of ten SAGE libraries from TS17 to TS26 (embryonic days 10.5-18.5) mouse pancreases as well as from adult islets and ducts. *Pdx1 *enhanced green fluorescent protein (EGFP) and *Neurog3 *EGFP reporter strains [22] were employed to allow fluorescence activated cell sorting (FACS) purification of pancreatic and endocrine progenitor cell populations, respectively, at early stages of mouse pancreas development. To our knowledge we are the first group to generate SAGE libraries from embryonic pancreas tissues. In sum, we sequenced over 2 million SAGE tags representing over 200,000 tag types, providing a truly comprehensive view of pancreas development. To validate our results, we assessed the temporal expression profiles of 44 genes by quantitative real-time PCR (qRT-PCR) and categorized the TS22 pancreas staining patterns of 601 genes in the *GenePaint *database [27,28], providing insight into the expression profiles of hundreds of transcripts previously not described in the pancreas. We then used the libraries to construct a network of predicted transcription factor interactions describing β-cell development, and validated selected linkages in this network using chromatin immunoprecipitation followed by qPCR (ChIP-qPCR) to detect enrichment of binding sites. Taken together, we anticipate these data will act as a framework for future studies on the regulatory networks driving pancreas development and function.

## Results

### Validating the biological significance of the pancreas SAGE libraries

In order to gain further insights into pancreas development and to provide a complementary analysis to available microarray data, we generated ten SAGE libraries from the mouse pancreas tissues by sequencing a total of 2,266,558 tags (Table 1). These libraries are publicly available at the Mouse Atlas [29] or CGAP SAGE websites [30] and can be analyzed using tools available through these sites. A total of 208,412 different tag types were detected in these libraries after stringent quality selection.

**Table 1:** Summary of pancreas SAGE libraries generated

Accession	Stage	Tissue subtype	Cell types represented	Library type	Tags sequenced*	Tag types
SM161/SM244	TS17	*Pdx1 *EGFP+^†^	All pancreas epithelial cells with the exception of rare Glucagon-positive cells	Long SAGElite	306,588	44,491
SM231	TS19	*Pdx1 *EGFP+	All pancreas epithelial cells with the exception of rare Glucagon-positive cells	Long SAGElite	317,716	49,572
SM162/SM245	TS20	*Ngn3 *EGFP-^†^	A mixture of pancreas cell types composed predominantly of mesenchymal cells and pancreas epithelial progenitors as well as those destined to become exocrine cell types	Long SAGElite	308,745	47,695
SM243/SM160	TS20	*Ngn3 *EGFP+	All endocrine progenitor cells as well as endocrine cells at various stages of maturation	Long SAGElite	320,473	51,847
SM225/SM249	TS21	*Ngn3 *EGFP+	All endocrine progenitor cells as well as endocrine cells at various stages of maturation	Long SAGElite	313,503	58,864
SM232	TS22	*Ngn3 *EGFP+	All endocrine progenitor cells as well as endocrine cells at various stages of maturation	Long SAGElite	301,222	37,726
SM223	TS22	Whole	A mixture of pancreas cell types composed predominantly of pancreas epithelial cells differentiating into exocrine cell types with some endocrine cells and mesenchymal cells	Long SAGE	98,189	13,676
SM016	TS26	Whole	A mixture of pancreas cell types composed predominantly of pancreas epithelial cells differentiating into exocrine cell types with some endocrine cells and mesenchymal cells	Long SAGE	81,130	17,963
SM102	DPN70	Isolated ducts	Hand picked adult ducts isolated by collagenase treatment and gradient centrifugation	Long SAGE	119,024	23,528
SM017	DPN70	Isolated islets	Hand picked adult islets isolated by collagenase treatment and gradient centrifugation composed of each of the major endocrine cell types	Long SAGE	99,968	16,039

*After 95% quality cutoffs for all tags. ^†^The *Pdx1 *EGFP and *Ngn3 *EGFP transgenic strains were obtained from Douglas Melton as described in Gu *et al*. [22]. DPN, days post natal.

**Figure 1:**
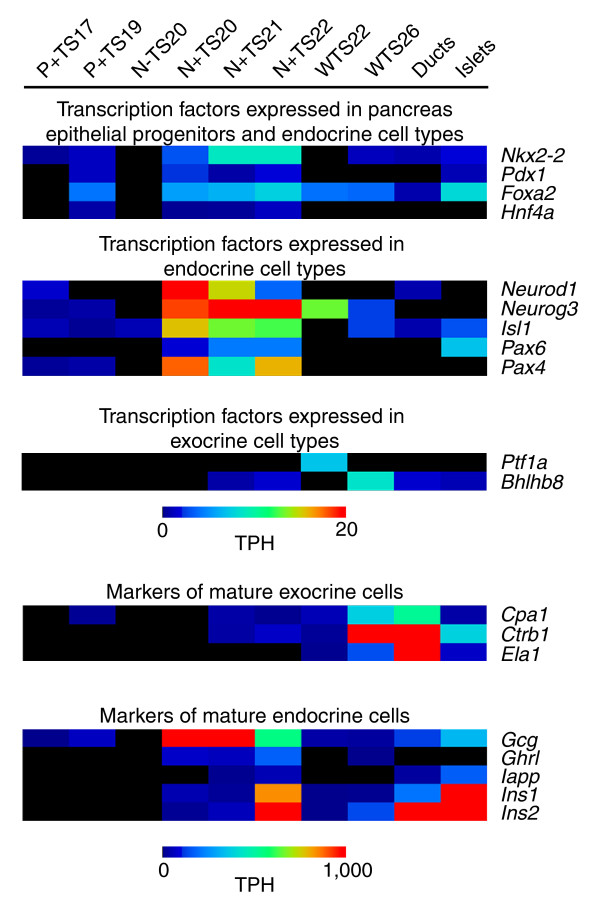
Heatmap of SAGE tag counts for genes with known expression profiles in pancreas development. Tags for genes with well characterized expression profiles in pancreas development were identified and their normalized counts obtained in each of the ten SAGE libraries created. A heatmap, generated using the multi-experiment viewer as described in the Materials and methods, of these results is shown based on the counts of the tags per hundred thousand (TPH). SAGE tags used include: TACACGTTCTGACAACT (*Nkx2-2*); AAGTGGAAAAAAGAGGA (*Pdx1*); TAGTTTTAACAGAAAAC (*Foxa2*); ACCTTCACACCAAACAT (*Hnf4a*); AATGCAGAGGAGGACTC (*Neurod1*); CAGGGTTTCTGAGCTTC (*Neurog3*); TCATTTGACTTTTTTTT (*Isl1*); GATTTAAGAGTTTTATC (*Pax6*); CAGCAGGACGGACTCAG (*Pax4*); CAGTCCATCAACGACGC (*Ptf1a*); AGAAACAGCAGGGCCTG (*Bhlhb8*); GACCACACTGTCAAACA (*Cpa1*); CCCTGGGTTCAGGAGAT (*Ctrb1*); TTGCGCTTCCTGGTGTT (*Ela1*); ACCACCTGGTAACCGTA (*Gcg*); GCCGGGCCCTGGGGAAG (*Ghrl*); CTAAGAATTGCTTTAAA (*Iapp*); GCCCTGTTGGTGCACTT (*Ins1*); TCCCGCCGTGAAGTGGA (*Ins2*). The libraries shown include: *Pdx1 *EGFP+ TS17 (P+ TS17); *Pdx1 *EGFP+ TS19 (P+ TS19); *Neurog3 *EGFP- TS20 (N- TS20); *Neurog3 *EGFP+ TS20 (N+ TS20); *Neurog3 *EGFP+ TS21 (N+ TS21); *Neurog3 *EGFP+ TS22 (N+ TS22); whole pancreas TS22 (WTS22); whole pancreas TS26 (WTS26); adult isolated ducts (Ducts); adult isolated islets (Islets).

To confirm that the libraries accurately represent the cell types intended (Table 1), we assessed the distribution of tags in the libraries for genes with well-characterized expression profiles in pancreas development. Figure 1 shows that transcription factors expressed in pancreas progenitor epithelial cells, such as *Pdx1 *and *Nkx2-2*, can be found in our TS17-TS19 *Pdx1 *EGFP+ libraries. Tags for these genes were also found frequently in the *Neurog3 *EGFP+ libraries. This is in agreement with the known expression of these factors. For example, *Pdx1 *is expressed in essentially all pancreas epithelial cells prior to the secondary transition while its expression after the secondary transition is abundant only in β-cells and β-cell precursors [8]. Prior to the secondary transition *Neurog3 *expression is quite low; however, at the start of the secondary transition its expression increases dramatically [31] and is subsequently lost quickly thereafter. This is precisely what we see in our data - low *Neurog3 *levels in the *Pdx1 *EGFP+ libraries, high expression in the *Neurog3 *EGFP+ libraries and diminishing expression in the TS22 and TS26 whole pancreas libraries, with no expression in the *Neurog3 *EGFP- or the adult islet or duct libraries. *Neurod1*, *Isl1*, *Pax6 *and *Pax4 *expression occurs subsequent to *Neurog3*, but unlike *Neurog3 *their expression is maintained in endocrine cell types [8]. In our data it is clear that the expression of all of these genes is most abundant in the *Neurog3 *EGFP+ libraries, or the islet library, as would be predicted. *Ptf1a *and *Bhlhb8 *(*Mist1*) are two transcription factors known to drive exocrine cell development. *Ptf1a *was found only in the TS22 whole pancreas library, and while low levels of *Bhlhb8 *were noted in the TS22 *Neurog3 *EGFP+ library, much higher levels were found in the duct cell library. Markers of mature exocrine cells showed peak expression in the TS26 whole pancreas or adult duct libraries, with moderate expression also in the islet library, suggesting a low level of exocrine cell contamination in this library. *Glucagon *expression peaked in the *Neurog3 *EGFP+ libraries, which is not surprising as Glucagon-positive cells are relatively abundant at these time points compared to in the adult islet. *Iapp*, *Ins1 *and *Ins2 *were all most abundant in the islet library, as was expected. The expression of these genes was also noted in the duct library, suggesting some level of islet cell contamination in this library. In sum, the expression profiles of these selected markers in our data match predictions based on their known expression profiles, indicating that our libraries accurately reflect the cell types and stages intended.

### Count and specificity thresholds

In SAGE data, tags with very low counts (especially those present as singletons) are enriched in error tags and their counts have little statistical power. It is useful, therefore, to use a minimum tag count threshold. To determine what count level to threshold our data at, in order to maximize the comprehensiveness of the data, while at the same time ensuring a high level of reliability, we assessed how different tag count thresholds affected the number of tags that mapped to known pancreas expressed transcripts or expressed sequence tags (ESTs). This analysis revealed that a threshold of a minimum raw count of 4 provided a good compromise between the number of tags kept and the percentage of tags that mapped to known pancreas expressed transcripts or ESTs (Additional data file 1). Additionally, in comparisons using Audic and Claverie statistics [32], tags with a count of 4 were statistically different from 0 at *p *≤ 0.05. From the 10 pancreas SAGE libraries, 16,233 tags met this threshold. Of these, 70% (11,656) mapped to known transcripts using the Refseq [33], Ensembl transcript [34], and MGC [35] databases with 85% (9,918) of these mapped unambiguously in the sense direction. These 9,918 unambiguously mapped sense tags represented 7,911 different genes, suggesting that many of the genes have alternative transcript termination sites, although this remains to be validated. A further 11% (1,817) of tags mapped only to the genome and possibly represent novel genes, leaving 17% (2,760) of tags we were unable to map. These results suggest the comprehensive nature of our data and suggest that our libraries are potentially a rich source of novel pancreas expressed transcripts.

It was of particular interest to us to identify genes with pancreas specific functions, rather than genes with ubiquitous roles in development or cellular function. We wanted, therefore, to institute a further threshold based on the specificity of the tags to the pancreas libraries. For this, we obtained the counts for the 11,735 tags that mapped unambiguously to a specific transcript or mapped uniquely to the genome in a total of 205 different SAGE libraries [36], including the libraries created here. Next, we calculated the specificities (S values) of each of these tags to each of the 205 libraries by dividing the ratio of the tag count in the library of interest versus its mean count in all the other libraries, multiplied by the log of its count in the library of interest, by the number of libraries the tag was found in. Tags were then ranked on their maximum specificity in any one of the pancreas libraries. Table 2 lists the 25 most specific tags identified in the pancreas libraries. As expected, tags that map to markers of mature pancreas cell types (that is, *Ins1*, *Ins2*, *Pnlip*) were very high on the list.

**Table 2:** Top 25 most specific transcripts in the pancreas SAGE libraries

Tag	Accession/location	Symbol	*Pdx1*-GFP+ (TS17)	*Pdx1*-GFP+ (TS19)	*Neurog3*-GFP- (TS20)	*Neurog3*-GFP+ (TS20)	*Neurog3*-GFP+ (TS 21)	*Neurog3*-GFP+ (TS22)	Whole (TS22)	Whole (TS26)	Ducts	Islets	MaxS^†^
TCCCGCCGTGAAGTGGA	NM_008387	*Ins2*	0*	0.31	0	13.11	57.43	3,298.9	4.07	139.28	1,422.4	2,2471.19	62.72
TTCTGTCTGGGCTTCCT	NM_023333	*2210010C04Rik*	0	0	0	0	0	0	0	77.65	651.97	109.03	33.43
GCCCTGTTGGTGCACTT	NM_008386	*Ins1*	0	2.83	0	45.56	19.59	839.25	6.11	9.86	207.52	3,116	27.90
TTAGGAGGCTGCTGCTG	NM_026925	*Pnlip*	0	0	0	0	0	0	0	0	1,760.99	116.04	18.10
CCCTGGGTTCAGGAGAT	NM_025583	*Ctrb1*	0	0.31	0	0	31.21	74.36	17.31	3,162.83	1,443.41	385.12	18.05
GCCCTGTGGATGCGCTT	NM_008387	*Ins2*	0	0	0	0	0.33	16.27	0	0	15.96	432.14	17.58
GTGTGCGCTGGTGGCGA	NM_007919	*Ela2*	0	0	0	0	0	0	0	69.03	181.48	4	11.75
GCATCGTGAGCTTCGGC	NM_007919	*Ela2*	0	0	0	0	0	2.32	0	1,329.96	2,680.13	1,156.37	11.24
GTGTGCGCCGGCGGCGA	NM_026419	*Ela3*	0	0	0	0	0	1	1.02	636.02	369.67	23.01	11.14
ACCACCTGGTAACCGTA	NM_008100	*Gcg*	7.5	63.26	0.65	2,554.97	1,952.71	550.42	34.63	25.88	124.34	326.1	10.99
AAAGTATGCAAATAGCT	NM_026918	*1810010M01Rik*	0	0	0	0	0	0	0	194.75	934.27	459.15	9.90
CAGACTAAGTACCCATA	NM_009885	*Cel*	0	0	0	0	0.66	1	0	750.65	375.55	16.01	8.81
TTTTACTTCTAAGAGTC	NM_021331	*G6pc2*	0	0	0	0.31	0	3.32	0	0	5.88	221.07	7.74
CCCGGGTGCAAGAAGAA	NM_018874	*Pnliprp1*	0	0	0	5.93	12.62	18.26	16.3	1,135.22	250.37	8	7.40
TCCCTTCAACCTTAGAC	NM_011271	*Rnase1*	0	0	0	0	0	0.33	0	221.87	1,249.33	170.05	6.48
TTAAACCAGAGTTCATA	NM_023333	*2210010C04Rik*	0	0	0	0	0	0	0	0	10.08	0	5.66
GCCTACAACTAAACTGT	NM_023182	*Ctrl*	0	0.31	0	0	0	0	0	27.12	491.5	195.06	5.46
GCACCAAGTACACATAT	NM_029706	*Cpb1*	0	0	0	0	0	0	0	303.22	209.2	21.01	5.11
TTGCGCTTCCTGGTGTT	NM_033612	*Ela1*	0	0	0	0	0	0	0	0	8.4	0	4.93
TGGGAGTGGAGGATGCC	NM_026925	*Pnlip*	0	0	0	0	0	0	0	0	29.41	9	4.83
TTCCAAGTGGAGGAGGT	NM_018874	*Pnliprp1*	0	0	0	0.31	0	0	10.18	163.93	36.97	1	4.78
CTAAGAATTGCTTTAAA	NM_010491	*Iapp*	0	0.31	0	3.43	6.64	49.8	0	2.47	25.21	170.05	4.50
CAGTCCATCAACGACGC	NM_018809	*Ptf1a*	0	0	0	0	0	0	7.13	0	0	0	4.36
CAAAGAATGCAATCTGA	nt_039700		0	0	0	0	0	0	7.13	0	0	0	4.36
CTTGCAGTCTGAGTTCG	nt_039413		0	0	0	0	0	0	7.13	0	0	0	4.36

*Tag counts are shown as tags per 100,000. This indicates the total number of times a given SAGE tag appears in the library per 100,000 tags and is used to normalize for libraries of varying size. ^†^S is the specificity of the tag. Specificity is calculated as described in the Materials and methods. The maximum S in any one of the libraries created here is indicated.

**Figure 2:**
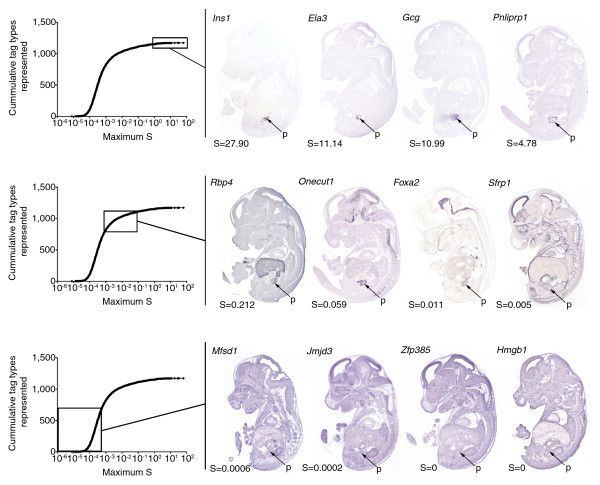
Specificity threshold accurately predicts spatial expression restriction. A plot of specificity (S) versus cumulative tag types represented shows the distribution of tags into tags with high (S > 0.1; top), medium (0.001 > S < 0.1, middle), and low (S < 0.001, bottom) S values. Representative *in situ *hybridization staining patterns from TS22 whole embryo saggital sections obtained from *GenePaint *are shown for each specificity group. Relevant *GenePaint *probe IDs can be found in Additional data file 4. Arrows indicate the location of the pancreas (p).

To validate that these rankings accurately reflect the level of restriction of a gene's expression pattern, we compared our results with TS22 whole embryo *in situ *hybridization staining patterns using the *GenePaint *database [27,28]. We did this with sets of transcripts with high (S > 0.1, representing 5% of the genes), medium (0.001 > S < 0.1, representing 25% of the genes), and low (S < 0.001, representing 70% of the genes) S values. Figure 2 indicates that the calculated S values correlated extremely well with the relative restriction of the staining seen in the TS22 whole embryo sections. Genes with high S values showed staining specifically in the pancreas, genes with medium S values showed staining in the pancreas and a limited number of other tissues, and genes with low S values showed broad staining throughout the embryo. Additionally, our metric met biological expectation and genes with known pancreas specificity (*Ins1 *S = 27.9, *Ins2 *S = 62.7, *Gcg *S = 10.985, and so on) had very high S values, while housekeeping genes (*Sdha *S = 0.0006, *HbS1L *S = 0.0002, *B2m *S = 0.0005) had very low S values. Meanwhile, genes with restricted expression to other tissues either did not meet our count threshold (*Plunc*, *Cldn13*, *Pomc*, *Prm2*, and so on) [37] or had very low S values (*Alb *S = 0.0007). Together, these observations provided confidence in our specificity metric and we set a threshold of a minimum S of 0.002, as this value occurs roughly at the inflection point between medium and high S values in the plot of S value versus cumulative tag types represented (Figure 2). In sum, 2,536 (approximately 20%) tags met this threshold.

### SAGE tag clustering

**Figure 3:**
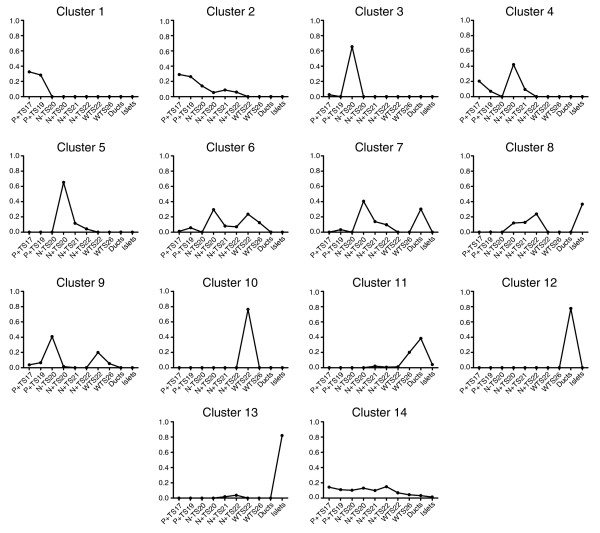
Median plots of identified SAGE tag *K*-means cluster analysis using 14 clusters. We clustered 2, 536 SAGE tags with a count greater than 4 in one of the SAGE libraries and with a minimum specificity of 0.002 and that map unambiguously to a specific transcript or genome location into 14 clusters using *K*-means clustering using a PoissonC algorithm as described in the Materials and methods. The median normalized tag counts for the tags in each of the clusters is shown plotted against the indicated SAGE libraries. The libraries shown include: *Pdx1 *EGFP+ TS17 (P+ TS17); *Pdx1 *EGFP+ TS19 (P+ TS19); *Neurog3 *EGFP- TS20 (N- TS20); *Neurog3 *EGFP+ TS20 (N+ TS20); *Neurog3 *EGFP+ TS21 (N+ TS21); *Neurog3 *EGFP+ TS22 (N+ TS22); whole pancreas TS22 (WTS22); whole pancreas TS26 (WTS26); adult isolated ducts (Ducts); adult isolated islets (Islets). A full list of the tags, the cluster they belong to, and their counts in each of the libraries is shown in Additional data file 2.

We next wanted to group the tags based on their differential expression during pancreas development so as to segregate them based on their potential functional significance to the different stages and cell types represented by our libraries. First, a FOM analysis for the *K*-means algorithm with Euclidean distance was performed on normalized data, essentially as described [38]. Based on these results we performed a 14-cluster analysis using the PoissonC algorithm [39] with subsequent hand curation to finalize the clusters (Figure 3 and Additional data file 2).

A summary of the clusters (Table 3) revealed that tags for genes with similar known pancreas function cluster together. For example, genes essential to endocrine cell specification were predominantly found in cluster 5, pancreatic enzyme genes in clusters 11 and 12, and islet hormone genes in cluster 13. The clusters also showed differential enrichment for Gene Ontology (GO) and Kyoto Encyclopedia of Genes and Genomes (KEGG) pathway terms (Table 3). Of interest, the clusters also had distinctively different median specificities, with cluster 5 containing genes with the highest median S, followed by cluster 13. These two clusters are enriched in genes in the mature onset diabetes of the young KEGG pathway and contain many endocrine specific factors, and this reflects the specialized nature of these cells. Cluster 14 had the lowest median S and the flattest expression profile of the clusters. In sum, these data suggested that the clusters represented biologically distinct gene sets.

**Table 3:** Summary of SAGE tag *K*-means cluster data

Cluster	Number of tags in the cluster	Number of genes in the cluster	Number of genome maps in the cluster	Number assessed by GenePaint*	Number assessed by QPCR	Median S^†^	Previously characterized genes in the cluster	Selected GO categories and KEGG pathways enriched in the cluster^‡^
1	154	85	61	37	3	0.0079	*Nkx6-2*	Transcriptional activator activity *p *= 0.02; development *p *= 0.049
2	49	29	19	15	2	0.0037		Metabolism *p *= 0.01; cell organization and biogenesis *p *= 0.035
3	58	40	15	14	2	0.0044		Receptor activity *p *= 0.028; development *p *= 0.030
4	292	115	175	45	4	0.00895	*Hes6*, *Pdx1*, *Sox9*	Regulation of transcription *p *= 0.027; maturity onset diabetes of the young *p *= 0.002
5	1,008	427	542	175	13	0.03555	*Arx*, *Gcg*, *Ghrl*, *Iapp*, *Isl1*, *Nkx2-2*, *Myt1*, *Neurog3*, *Neurod1*, *Pax4*, *Pax6*, *Pou3f4*, *Pyy*	Secretory pathway *p *< 0.001; hormone activity *p *= 0.049; maturity onset diabetes of the young *p *< 0.001
6	60	41	17	16	1	0.00465		
7	21	11	10	7	1	0.008		
8	78	46	28	29	5	0.012	*Pax6*	Eye morphogenesis *p *= 0.020; type II diabetes mellitus *p *= 0.001
9	23	16	6	10	2	0.0041		Cell proliferation *p *= 0.028
10	401	281	107	122	4	0.0158	*Id2*	Response to endogenous stimulus *p *= 0.021
11	76	57	10	23	1	0.00555	*Amy1*, *Cel*, *Clps*, *Ela1*, *Pnliprp2*, *Reg1*	Protein catabolism *p *= 0.002
12	154	122	13	56	3	0.0074	*Ela1*, *Pnlip*, *Reg3d*	Growth factor binding *p *= 0.005; carboxypeptidase activity *p *= 0.013; regulation of cell growth *p *= 0.027
13	136	84	42	30	3	0.01835	*Iapp*, *Ins1*, *Ins2*	Secretion *p *= 0.03; maturity onset diabetes of the young *p *< 0.001; type II diabetes mellitus *p *< 0.001; type I diabetes mellitus *p *= 0.003
14	56	47	4	22	0	0.00335		Protein metabolism *p *= 0.020

*Refers to the number of genes analyzed by *in situ *hybridization using *GenePaint *[62] on TS22 whole embryo cryo-sections that gave informative staining. ^†^S is the specificity of the tag. Specificity is calculated as described in the Materials and methods. ^‡^GO term enrichments and *p*-values were calculated using EASE while KEGG pathway enrichments and *p*-values using Webgestalt as described in the Materials and methods.

### Validation of SAGE tag clusters

To validate the identified clusters, we first compared our data to lists of genes determined to be enriched in pancreatic progenitors, endocrine cells, or islets using Affymetrix microarray analysis of *Pdx1 *EGFP+ and *Neurog3 *EGFP+ cells and islet tissues, similar to those used here [22]. There were 107 genes present in both genes sets and the representation of each enrichment group from the array analysis in our clusters calculated (Additional data file 3). Of the 29 genes identified as enriched in pancreatic progenitors in the microarray analysis, we identified 13 of these in clusters 1-3 or cluster 9 that show peak expression early in pancreas development. Another 11 were found in clusters 10 and 11 that show peak expression in the TS26 whole pancreas library or the duct library, stages and tissue types that were not used in the array analyses. Of 24 genes identified in the array study as enriched in endocrine cells, 19 were found in cluster 5, with 2 more in cluster 4, both of which show peak expression in the *Neurog3 *EGFP+ libraries here. Of the genes identified as islet enriched in the array studies, 16 of 54 were classified as such in our study; a further 20 were found in clusters 11 and 12 that have peak expression in the ducts, again a tissue not represented in the array studies; and a further 10 were found in clusters 5 or 8 that show peak expression in the *Neurog3 *EGFP+ libraries and islet library, respectively. Overall, the two data sets compare well and the majority of genes were identified as enriched in the same cell populations, although the differences in the tissues used in each study, specifically our inclusion of developing whole pancreas and adult duct libraries, did cause differences in some of the results.

**Figure 4:**
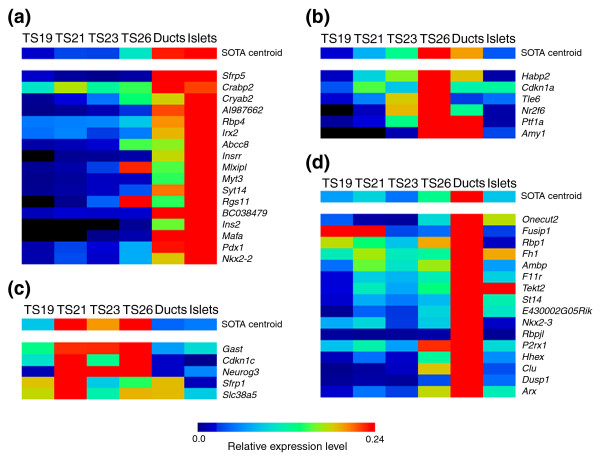
SOTA clustering of temporal expression profiles from qRT-PCR analysis of 44 genes in pancreas development. qRT-PCR was used to determine the relative expression levels of the indicated genes during pancreas development at the TSs indicated. The relative level of expression of each gene was normalized and a SOTA analysis used to group the genes. Heatmaps of the relative expression levels of the genes in the SOTA groups, including the SOTA centroid, with peak expression in **(a) **the islets, **(b) **the TS26 developing pancreas, **(c) **the TS21-TS26 developing pancreas, or **(d) **the ducts are shown. The data shown are averages of the results obtained from pancreases from three separate litters (pancreases from an individual litter were pooled) or islet/duct collections with triplicate reactions from the separate RNA extractions.

To further confirm that our clusters accurately group genes with similar temporal expression profiles, we analyzed the expression of 44 genes through pancreas development using qRT-PCR. Selected targets included *Ins2*, *Nkx2-2*, *Pdx1*, *Neurog3*, *Amy1*, and *Ptf1a*, which all have well established expression profiles as references. We then used a self-organizing tree algorithm (SOTA) clustering analysis to group the obtained temporal expression profiles for these genes. This allowed us to determine if groupings similar to those found in the SAGE data cluster analysis were observed. In our SOTA analysis, genes with four distinct expression profiles were identified (Figure 4): one group with peak expression in the islet sample, one with peak expression in the TS26 whole pancreas, one with peak expression from TS21-TS26, and one with peak expression in the ducts sample. All of the genes in the SOTA groups containing *Ins2*, *Mafa*, *Pdx1*, and *Nkx2-2*, which are markers of the endocrine lineage, were from clusters 1, 4, 5, and 13. Three of the six genes in the SOTA group with peak expression at TS26 were from clusters 4 and 5, although each of these showed relatively high expression in either the TS22 or TS26 whole pancreas libraries. Of the genes in the SOTA group with peak expression from TS21-TS26, one was from cluster 3, two were from cluster 5 and one was from cluster 9. Clusters 3 and 9 are enriched in mesenchymal factors (see below). Since no mesenchymal cells should be present in the islet and duct samples, it makes sense for these genes to have this expression profile. Two genes from cluster 5 were in this SOTA group, including *Neurog3*, which is known to be developmentally restricted in expression, and *Gast*, likely reflecting the relative number of Gastrin-producing cells in the different samples. Of the 11 genes in the SOTA group with peak expression in the ducts sample, 4 were from clusters 7 and 12, while the rest were found in the other clusters, although significantly excluding clusters 13 and 8. All of the genes in this group had counts in the duct library, despite being in clusters with peak expression in other libraries, although they all had, in general, low overall tag counts.

### *GenePaint *analysis

**Figure 5:**
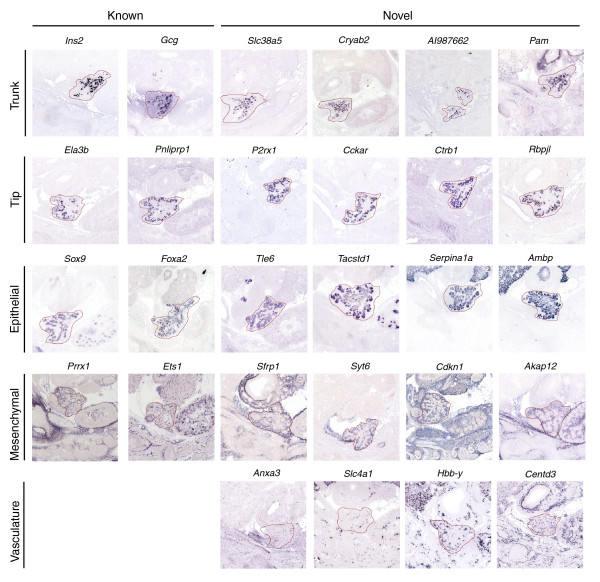
Representative *in situ *staining patterns for genes expressed in each of the identified expression profiles. Representative genes for each of the identified spatial expression profiles, including genes with known and previously un-described, or novel, staining profiles in pancreas development, are shown. For this, images of *in situ *hybridization staining patterns for whole embryo sagittal sections were obtained from the *GenePaint *website and magnified to show the pancreas (outlined in red). Relevant *GenePaint *probe IDs can be found in Additional data file 4.

Taken together, the data suggested that the generated clusters represent transcript sets with distinct roles in pancreas development. To further confirm this, we assessed whether the transcripts identified in each of the SAGE tag clusters had spatial expression profiles consistent with these roles using the *GenePaint *database [27,28]. For each of the 923 genes present in our clusters and in the *GenePaint *database, we analyzed the *in situ *hybridization staining pattern in the pancreas from TS22 whole embryo sections. In sum, 601 of the genes showed informative staining, and these were categorized based on their staining patterns into one of five expression domains found in the pancreas [40] (Figure 5). For the remaining 316 genes, either the probes did not show stain in any sections or sections with pancreas were not present in the database. Regardless, we identified 88 genes expressed in the tips of epithelial branches that at E14.5 primarily contain exocrine progenitor cell types. A further 81 genes were identified as expressed in the trunk of the epithelial branches that contains endocrine and ductal progenitor cells; 221 genes were identified as expressed throughout the epithelium; and a further 51 were found only in the mesenchyme, and 42 in the vasculature. For a full categorization of the genes see Additional data file 4. There were 124 (13%) genes identified in our SAGE data that were not detected in the pancreas at the time point assessed. The average tag count for these genes was only 6.8 while for detected genes it was 24, suggesting this is, in part, due to the low expression levels of these genes. Moreover, the number of genes not detected was highest in clusters 1, 2 and 13, which include genes that show low relative expression at TS22.

**Figure 6:**
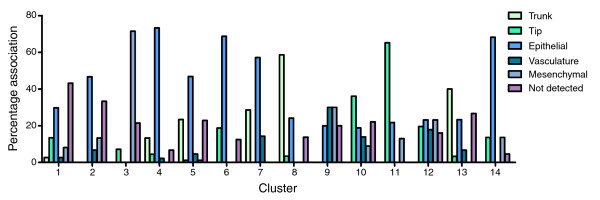
The percent association of genes in each *K*-means cluster with the five expression domains in the pancreas. The *in situ *hybridization staining profiles of 605 genes with informative stain in TS22 pancreas tissue were classified into the groups shown using the *GenePaint *database. Additional data file 4 lists the full categorization of each of these genes. The percentage of genes with each staining profile in each of the SAGE tag *K*-means cluster is shown.

Analysis of the representation of each of the five staining profiles in the 14 identified SAGE tag clusters (Figure 6) revealed that some clusters are far more predictive of specific expression profiles than others. For example, clusters 9 and 12 were not at all predictive of a given staining pattern. In contrast, 73% of the genes analyzed in cluster 4 showed pan-epithelial staining, while 59% of those in cluster 8 showed trunk staining and a further 24% showed pan-epithelial staining. These data suggest that several of the clusters represent genes with distinct spatial expression profiles. Significantly, these profiles are consistent with the known roles of genes within the clusters. For example, cluster 5, which is enriched in genes involved in mature onset diabetes of the young, contains tags with peak expression in the *Neurog3 *EGFP+ libraries and genes in this cluster predominately show pan-epithelial or trunk expression. It is apparent from these results, in combination with the median profiles of the clusters (Figure 3), that clusters 1, 2, and 4 represent genes appropriately expressed spatially and temporally to be functionally significant in pancreatic progenitor cells, while genes in clusters 4, 5, and 8 are likely functionally significant in endocrine progenitors, genes from clusters 6, 10, and 11 in exocrine progenitors, genes from cluster 3 in mesenchymal cells, and genes from clusters 8 and 13 in adult islet cells. Taken together, these analyses allowed us to identify lists of transcripts, many of which have not previously been characterized in pancreas development, that are appropriately expressed spatially and temporally to play functionally significant roles in each of the major phases of pancreas development.

### Identification of a transcriptional cascade in endocrine pancreas development

**Figure 7:**
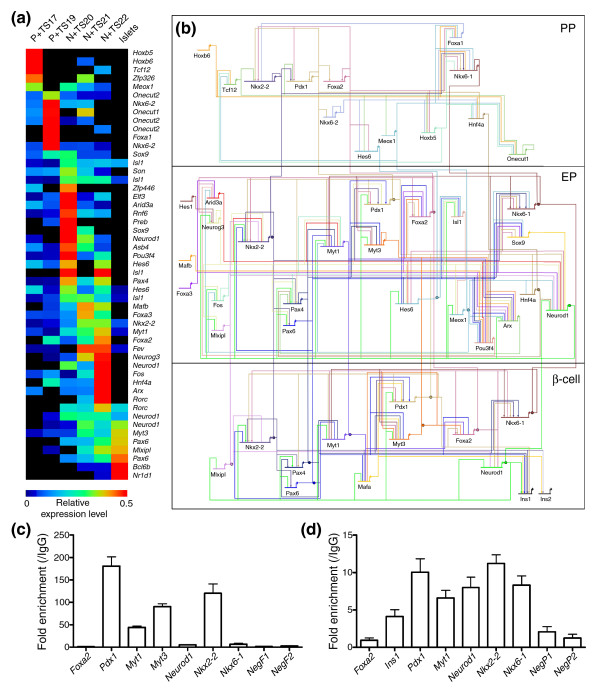
A cascade of transcription factors expressed in endocrine cell development. Tags that mapped unambiguously to transcription factors and met our count and specificity thresholds were identified and their normalized counts obtained in each of the SAGE libraries that represent the development of the endocrine lineage. **(a) **A heatmap, generated using the multi-experiment viewer as described in the Materials and methods, of these results is shown. Tags are organized by the order of their expression in the libraries. The libraries shown include: *Pdx1 *EGFP+ TS17 (P+ TS17); *Pdx1 *EGFP+ TS19 (P+ TS19); *Neurog3 *EGFP+ TS20 (N+ TS20); *Neurog3 *EGFP+ TS21 (N+ TS21); *Neurog3 *EGFP+ TS22 (N+ TS22); adult isolated islets (Islets). **(b) **A transcription factor network in β-cell development. The network was generated as described in the materials and methods using Biotapestry to visualize the network [65]. Ins1 and Ins2 are shown as the final products expressed in mature β-cells. Literature reported interactions [8,41] are included with arrow heads indicating positive regulation and perpendicular lines repression. Interactions in pancreatic progenitors (PP), endocrine progenitors (EP) and mature β-cells are shown. **(c) **The fold enrichment of predicted Foxa2 binding sites, as detected by qPCR, in the promoters of *Pdx1*, *Myt1*, *Myt3*, *Neurod1*, *Nkx2-2*, *Nkx6-1*, and of two sites not predicted to contain a Foxa2 site (*NegF1 *and *NegF2*) in ChIP experiments using an anti-Foxa2 antibody compared to IgG. **(d) **The fold enrichment of predicted Pdx1 binding sites, as detected by qPCR, in the promoters of *Foxa2*, *Ins1*, *Myt1*, *Neurod1*, *Nkx2-2*, *Nkx6-1*, and of two sites not predicted to contain a Pdx1 site (*NegP1 *and *NegP2*) in ChIP experiments using an anti-Pdx1 antibody compared to IgG. The data shown are averages of the results obtained from three separate ChIP experiments each with duplicate reactions. Error bars indicate the standard deviation of the averaged replicates.

Transcriptional regulators, in concert with signaling factors, provide the genetic instructions that drive endocrine pancreas development. Based on our analyses, genes in the endocrine lineage (that is, from pancreatic progenitor cells through endocrine progenitors to adult islet cells) can be identified based on their presence in clusters 1, 2, 4, 5, 8 and 13. We identified 58 tags for transcriptional regulators in these clusters representing 43 different factors. Eliminating factors only expressed in one library, and for which we could not find additional support for their expression, left 38 different factors for which there is good evidence of their expression in the endocrine pancreas lineage. Eight of these genes were represented by multiple tag types, with four separate tags mapping to *Neurod1 *and *Isl1*. Figure 7a shows a heatmap of the expression of these factors in endocrine cell development as detected in our SAGE data. Many of these factors have well-established roles in pancreas development, including *Ngn3*, *Pax4*, *Nkx2-2*, *Pdx1*, *Isl1*, and *Neurod1*. However, several factors with uncharacterized roles were also identified, including: *Tcf12*, *Zfp326 *and *Meox1*, which were most abundant in *Pdx1 *EGFP+ cells; *Zfp446*, *Rnf6*, and *Son*, which were most abundant in *Neurog3 *EGFP+ cells; and *Nr1d1*, *Myt3*, and *Bcl6b*, which were most abundant in islet cells.

The transcriptional circuits essential to β-cell development are only beginning to be elucidated [8,41] and the majority of regulatory interactions driving pancreas development remain unknown. Expression profiling has been used extensively to generate networks with co-expressed genes 'linked' using the assumption that co-expression implies co-regulation [42-45]. The promoters of co-expressed genes are often then analyzed for common sequence elements and the presence of known binding sites. One advancement on this technique is to include analysis of sequence conservation under the elements to better predict 'real' sites [46]. Such methods have been successfully applied to mammalian cells to identify networks active in B-cells and during macrophage activation [44,45,47]. Therefore, to similarly gain further insight into β-cell development, we utilized our transcription factor cascade as it represents sets of co-expressed transcription factors.

For this, we first eliminated factors for which we could not find direct evidence of expression in the cell types in the β-cell lineage, that is, pancreas progenitors, endocrine progenitors, and mature β-cells, using the *GenePaint in situ *data, literature, or the EPConDB/T1Dbase [48], or as mentioned above were found in only one library. We then searched the proximal promoters of each of the remaining transcription factors, using the CisRED database of highly conserved sequence elements in the proximal promoters of genes from multiple species [49], for binding sites of co-expressed transcription factors or of factors expressed in the parent cell type. From this we generated predicted transcription factor interaction lists. Overall, our predictions identified 28% of transcription factor interactions reported in the literature [8], with some binding sites less predictive than others. Randomly generated interaction lists using the same transcription factors identified only 3% of literature reported sites, indicating that our interaction predictions are significantly enriched for true sites (*p *= 5.7 × 10^-6 ^using Fisher's exact test). These predicted interactions, as well as literature reported interactions, were then used to build a transcriptional network describing endocrine pancreas development (Figure 7b). In sum, a total of 217 novel predicted interactions are shown in this network, suggesting that it is a rich source of hypotheses for future validation. To validate this and to gain an understanding of the false positive rate associated with our interaction predictions, we utilized ChIP-qPCR to validate predicted targets of Foxa2 and Pdx1 in the β-cell portion of the network using Min6 cells. For Foxa2 we found high levels of enrichment for predicted sites in the promoters of *Pdx1*, *Myt1*, *Myt3*, and *Nkx2-2*, but not for predicted sites in the promoters of *Foxa2*, *Neurod1*, or *Nkx6-1 *(Figure 7c). For Pdx1 modest levels of enrichment were achieved for sites in the promoters of *Ins1*, *Pdx1*, *Myt1*, *Neurod1*, *Nkx2-2*, and *Nkx6-1*, although no enrichment was seen for a predicted site in the *Foxa2 *promoter (Figure 7d). No enrichment was seen at either of two sites that were not predicted to contain a binding site for either Foxa2 or Pdx1. In total, 10 out of 14 (71%) predicted interactions proved valid, with 4 of 7 (57%) validating for Foxa2 and 6 of 7 (86%) for Pdx1.

## Discussion

Taken together, our data suggest that the pancreas libraries generated and analyzed in this study accurately reflect the cell types intended and are highly comprehensive. Our analysis of the tag distribution of genes with well characterized expression profiles in the libraries confirmed their known expression. As well, 79% of the genes we identified, after instituting a count threshold, have independent validation of pancreas expression via EST information. Of the genes we analyzed using the *GenePaint *database, 20% were not detected, comparable with the expected detection rate of this technique, with this number as low as 13% for clusters with expression at TS22, the time point analyzed. It has been suggested that a SAGE library of 120,000-160,000 tags is roughly equivalent in sensitivity to a microarray [50]. Six of the ten libraries generated here were sequenced to greater than 300,000 tags per library with the remaining four sequenced to approximately 100,000 tags per library. It is clear that these libraries are a rich source of information on the expression profiles of a large diversity of known and unknown transcripts throughout pancreas development. Interestingly, multiple tags were identified for many genes with critical roles in pancreas development and function, including *Ins1*, *Ins2*, *Isl1*, *Neurod1*, and *Pax6*; the biological relevance of this remains to be elucidated. Additionally, we identified 1,049 tags that meet both our count and specificity thresholds and mapped only to the genome. Although some of these tags are likely to be error tags, they represent a rich source of information on completely novel transcripts.

To fully appreciate the data generated in this study it is important to consider the cell types the libraries represent. Two of the libraries generated use a transgenic mouse strain that expresses EGFP under control of the *Pdx1 *promoter, while four libraries were generated using a transgenic strain expressing EGFP under control of the *Neurog3 *promoter [22]. Prior to the secondary transition, *Pdx1 *marks all pancreas epithelial cells, with the exception of rare glucagon-expressing cells. At the stages used here, TS17 and TS19, the collected cells therefore represent a relatively pure population of pancreas epithelial progenitor cells, which will eventually give rise to all subsequent endocrine and exocrine cell types in the pancreas. *Pdx1 *is also expressed in the duodenum at these time points and some level of duodenum specific transcripts may also be present, although care was taken in the dissections to minimize this. *Neurog3 *is extremely specific in its expression and in the pancreas is exclusively expressed in endocrine precursors, the cells that give rise to all of the hormone-producing cell types in the pancreas. *Neurog3 *expression itself is transient and decreases substantially after TS23 [17]. However, the EGFP protein is relatively stable and continues to mark endocrine cells as late as TS26 [26]. Thus, the *Neurog3 *EGFP+ libraries collected here, from TS20 to TS22, represent a mixture of endocrine progenitor cell-types, predominantly α and β cell precursors, in various stages of differentiation and maturation. The *Neurog3 *EGFP- TS20 library contains a variety of pancreas cell types, including mesenchymal cells, as well as epithelial and exocrine progenitors. Similarly, the TS22 and TS26 whole pancreas libraries are composed of various cell types, at TS22 predominantly mesenchymal cells and various epithelial cell types, including endocrine and exocrine progenitors, while at TS26 a greater relative abundance of exocrine cells is found. The Islet and Duct libraries represent fully mature cell types. These libraries were constructed from hand-picked islets and ducts isolated by collagenase digestion and gradient centrifugation and, as such, contain a minimum of contaminating exocrine cells. The Islet library is predominantly composed of β-cells that compose approximately 80% of a rodent islet [51], with α-cells composing much of the remaining cells, with smaller numbers of δ, ε, and PP cells also present. For the Duct library, epithelial cells from duct tips were collected, which have been proposed to harbor pancreas stem cells [52], although transcripts from contaminating exocrine and islet cells were inevitably identified as well.

One of the major strengths of SAGE data is the ability to easily compare data amongst different libraries. We took advantage of this strength and were able to institute a specificity-based threshold for our data by obtaining the counts for tags that met our count threshold in 205 different SAGE libraries. In general, metrics for specificity assess the level of expression in the tissue of interest versus the total expression. One study used a metric based on Shannon entropy to account for bias from differing levels of expression in different tissues [53]. This metric is not, however, directly applicable to SAGE data due to the large number of tags with no counts in many samples. Here we use a metric that accounts for the relative expression of a tag, the number of libraries it is found in, as well as its absolute expression level. Thus, tags expressed at high-count levels in the library of interest and with low levels in very few other libraries have the highest specificity, while tags with low counts in numerous samples have the lowest specificity. We show that this metric performs well and that it accurately identifies genes with relative levels of restriction compared with available *GenePaint *data, and meets biological expectation.

The use of a specificity threshold to assess data from transcriptome-based approaches to pancreas development is novel and previous studies have relied on the identification of genes of interest based on their differential expression in pancreas development. However, differential expression can occur for many reasons and many of the tags that did not meet our specificity threshold showed large changes in count levels between the libraries (data not shown), suggesting that assessing differential expression alone does not screen out many widely expressed genes and that the addition of a specificity threshold is helpful in producing gene lists with the highest functional relevance. For example, of 30 genes identified as being the most significantly differentially expressed from TS21-TS25 in *Neurog3 *EGFP+ cells using the PancChip 6 [26], 10 did not make our count cut-off and 15 did not make our specificity cut-off. Additionally, with a few exceptions - tags for *Mafa *were found at a count of only one in the islet and TS22 *Neurog3 *EGFP+ library and, thus, missed our count threshold - almost all of the transcription factors known to play significant roles in pancreas development were present on our lists. Thus, by instituting our count and specificity thresholds we feel we have generated a tag list more enriched for genes of biological significance to pancreas development and function than previously accomplished.

Our data in general confirm the accuracy of our SAGE tag clustering. In our qRT-PCR validations, 10 of the 44 genes assessed had a maximum tag count of only 10 and our qRT-PCR data did not match the SAGE data as well, for these genes, as it did using genes with higher tag counts. Thus, some caution in interpreting the expression profiles of transcripts with low tag counts is necessary. Regardless, it was clear that tags with even very low counts were still indicative of actual expression. Clearly, deeper sequencing, particularly of the libraries at 100,000 tag depth, would improve the statistical power of the observed expression differences and provide a more robust detection of rare transcripts.

Five distinct domains of expression have been identified in the developing pancreas [40]. Although, several factors expressed in endocrine cells, which are found in the epithelial trunk, have been identified, relatively few factors are known to be expressed in the remaining expression domains [8,40]. Here we identify and categorize into the 5 expression domains the expression profiles of over 400 genes in the developing pancreas. In sum, we provide spatial and temporal expression data on hundreds of transcripts not previously characterized in pancreas development. Combining our SAGE tag clustering and *GenePaint *data provides a clear indication as to the stages of pancreas development at which many of these genes are likely functionally significant. This, and studies on retinal development [54], demonstrate the power of combining SAGE tag clustering with large scale *in situ *hybridization data.

Further, we utilized predicted and literature reported binding data to gain insight into the cascade of transcription factors that drives endocrine pancreas development. As already mentioned, our predicted interactions identified 28% of literature reported sites. The CisRED database used to generate our interaction lists identifies conserved sequence elements only in proximal promoters (approximately 1 kb upstream). Of course, not all regulatory elements are in proximal promoters and this is likely a major reason a higher percentage of known interactions was not identified. Future experimentation to locate and analyze regulatory elements across the genome will better predict all possible interactions. Optimally false positive error rates in such networks are calculated using comparisons with comprehensive binding data for multiple factors. However, this type of data is not available for the relevant transcription factors in pancreas tissues. We therefore attempted to gain an understanding of our false positive rate by utilizing ChIP-qPCR to validate predicted binding sites for Foxa2 and Pdx1. Although limited in scale, these validation experiments give us a reasonable estimation of our false positive rate as approximately 29%. However, this error rate varied significantly from 14% for Pdx1 to 46% for Foxa2. In part, this is due to our reliance on available binding data for the different transcription factors in the network, and thus the reliability of the predicted interactions is highly dependent on the quality of the available binding data, which varies significantly. It is also possible that some of the interactions we tested do not occur in the cell line used, but do in fact occur *in vivo*, although this remains to be tested. Regardless, it is a given that better binding data for a greater number of transcription factors are essential to the construction of more accurate networks in this fashion. Despite this, it is clearly of great interest to begin to elucidate the relevance of many of these predicted interactions in pancreas development and function.

## Conclusion

Previous studies have used microarrays to assess expression profiles in the developing pancreas. Here we utilized SAGE to provide a more comprehensive and complementary analysis, and to yield further insights into signals critical in regulating pancreas development. By instituting a specificity threshold for our data we have isolated a list of tags highly enriched for genes with significant roles in pancreas development and function. Equivalent analyses have not been performed in previous array studies, and highlight one of the strengths of SAGE as such comparisons are relatively straightforward with this technique. Our data represent a highly comprehensive analysis of pancreas development. Our classification of the *in situ *staining patterns of over 400 genes, in combination with our cluster analysis, allowed us to identify gene sets describing each of the major stages of pancreas development represented by our libraries. These data provide evidence for the pancreas enriched expression of hundreds of factors that have not previously been implicated in pancreatic development, suggesting that our data are a rich source of genes with novel roles in pancreas development. Further, from our data we constructed a predictive transcriptional network driving endocrine pancreas development that predicts many novel interactions that are of great interest for future analysis. In sum, these data provide insight into the regulatory networks driving pancreas development and function and provide a framework for future studies.

## Materials and methods

### Mouse maintenance and islet isolation

All mice were bred and maintained at the British Columbia Cancer Research Centre animal facility according to the guidelines of the Canadian Council on Animal Care. All protocols were approved by the University of British Columbia Animal Care Committee. Mice were housed in micro-isolator units, provided with Purina mouse food and autoclaved water *ad libidum*, and were maintained at 20°C ± 2°C under a light/dark cycle (light, 05:00-19:00; dark, 19:00-05:00). Males were mated overnight with up to three females and females were checked for plugs before 9:30 the following morning. Plugged mice were considered to be 0.5 days post coitum.

### Generation of SAGE libraries

Embryos, obtained from timed pregnant female *Pdx1 *EGFP and *Ngn3 *EGFP mice mated with C57Bl/6J mice, were staged using Theiler criteria. Embryos were collected in ice cold phosphate buffered saline (PBS) solution. The developing pancreas was isolated by micro-dissection while the embryo was submerged in PBS. For TS17-TS22 *Pdx1 *EGFP and *Ngn3 *EGFP FACS sorted libraries, a single cell suspension was generated by Trypsin/EDTA digestion. The sample was prepared for sorting by staining with propidium iodide and passing through a 40 μm filter with a final resuspension in FACS buffer (PBS containing 2% fetal bovine serum and 2 mM EDTA). EGFP positive or negative cells were sorted and collected as appropriate into a microcentrifuge tube containing Trizol^® ^using BD FACSVantage instruments (operated by the Terry Fox Laboratory at the BC Cancer Research Centre). Between three and eight litters of mice were used to generate each library, depending on the stage. Islets and ducts from at least 10 separate mice were purified from 8-10-week-old ICR males by collagenase digestion and gradient centrifugation as previously described [55] and placed in Trizol^®^. Total RNA was extracted using Trizol^® ^and RNA quality was assessed using a Bioanalyser (Agilent, Santa Clara, CA, USA). For the TS22 and TS26 whole pancreas as well as the islet and duct libraries roughly 10 μg of total RNA was used to construct each SAGE library. For the FACS sorted libraries a SAGElite approach [56] was used with a minimum of 20 ng of total RNA. All libraries were constructed as previously described [36].

### SAGE data analysis

SAGE data were analyzed using DiscoverySpace4 [57]. All SAGE libraries were generated as part of the Mouse Atlas of Gene Expression project [36] and filtered for sequence quality so that each tag had a 95% or greater probability of being correct, using the PHRED score quality assessment software [58]. Tag to gene mapping was performed using the mouse Refseq, MGC, and Ensembl databases using the DiscoverySpace program. Tags were considered sense position matches if they mapped in the sense orientation to the gene and antisense matches if they mapped in the opposite orientation. A tag was considered unambiguous if it matched a single sense position gene in all of the databases, and ambiguous if it mapped to multiple genes in a sense position regardless of the mapping position.

The specificity of tags was determined by first obtaining the counts for the tags in 205 different Mouse Atlas libraries. From this the mean of the tag counts in all the libraries (M_a_) was determined and compared to tag count in the library of interest (C_i_) to obtain the mean ratio (M_r_). The total number of libraries the tag was found in, or library count, was next determined (L_a_) as was the total counts of the tag in the library under analysis (C_i_). The specificity (S) was then calculated as:

S = M_r _log_1.3_(C_i_)/L_a_

Thus, tags with a high mean ratio that appear in relatively few libraries and are expressed more abundantly will have the highest specificities.

In short, the data were first normalized and FOM analysis for the *K*-means algorithm with Euclidean distance was performed on normalized data using 5 iterations and a maximum of 20 clusters. For this we used the multiexperiment viewer from the Institute for Genomic Research [59]. From this a cluster number of 14 was chosen and used in a PoissonC-based clustering strategy specifically designed for SAGE data [39]. GO term comparisons using EASE [60] and KEGG pathway enrichments were calculated using Webgestalt [61], comparing against the Webgestalt mouse database using a hypergeometric test to calculate *p*-values and a minimum of two genes required in a gene set. Heatmaps were generated using relative tag abundances using the multiexperiment viewer from the Institute for Genomic Research [59].

### *GenePaint *analyses

Images of *in situ *hybridization staining patterns for whole embryo sagittal sections were obtained from the *GenePaint *website [28,62] for 923 genes identified in our clusters. Additional data file 4 lists the gene names and *GenePaint *probe IDs for all of these genes. When appropriate, higher magnification images of the area of the embryo containing the pancreas were obtained and the pancreas outlined. The brightness and contrast of some of the images were altered using Photoshop to better assess the staining pattern. Genes were classified as showing trunk, tip, epithelial, mesenchmymal or vasculature staining, based in part on the similarity of their staining pattern to staining patterns for *Ins2*, *Gcg*, *Neurog3*, *Ela3*, *Pnliprp1*, *Foxa2*, *Sox9*, *Prrx1*, or *Ets1*.

### Quantitative real-time PCR

Probes for *Ins1*, *Ins2*, *Pdx1*, *Neurog3*, *Ptf1a*, *Nkx2-2*, *Amy*, and *β-actin *were purchased from Applied Biosystems (Foster City, CA, USA). All other primers were designed using Primer3 [63] and spanned introns where possible (Additional data file 5). Amplicons were between 80 and 120 bp for efficient amplification. Primer efficiencies were determined using a dilution series of TS24 cDNA. Only primer pairs with an efficiency greater than 0.8 were used in subsequent analyses. An ABI 7500 real-time PCR system (Applied Biosystems) and SYBR^® ^Green supermix (Applied Biosystems) or Universal PCR Master Mix (Applied Biosystems) was used for all reactions. Triplicate cDNAs were obtained by reverse transcription of 1 μg of total RNA from newly isolated pancreas tissue for each reverse transcription. Generated cDNA (10 ng) was used in each reaction with all reactions done in duplicate. Samples were normalized to GAPDH, and the fold increase compared to TS23 as calculated using 2^-ΔΔCt^ [64]. To determine significance, the non-parametric Mann Whitney test was used to compare the ΔCt values of the reference and the sample using Prism4 (GraphPad Software, La Jolla, CA, USA). SOTA analysis was performed using the multiexperiment viewer from the Institute for Genomic Research [59] using Euclidean distance, 4 max. cycles and default parameters.

### Network generation

To generate networks, transcription factors with evidence of expression in the relevant cell types in addition to being in our SAGE data (in at least two libraries to eliminate possible artifacts), from the literature or in the EPConDB/T1Dbase [48] were identified. The binding site preferences of the identified transcription factors were then obtained from Transfac or the literature (Additional data file 6). These sites were then used to search the CisRED database [49] to identify conserved motifs in the proximal promoters of factors that were co-expressed or expressed in daughter cells. This allowed us to create predicted transcription factor interaction lists, with literature reported interactions layered on these predictions [8,41]. In cases where two factors with the same binding site were co-expressed - for example, Neurog3 and Neurod1 - both are shown to be linked to the target site. The resulting network was then visualized using Biotapestry [65].

### Chromatin immunoprecipitation

Min6 cells were homogenized in 1% formaldehyde and incubated at room temperature for 10 minutes, prior to the addition of 0.125 M glycine followed by incubation at room temperature for 5 minutes. Cells were pelleted and resuspended in 5 volumes ChIP cell lysis buffer (10 mM Tris-Cl, pH 8.0, 10 mM NaCl, 3 mM MgCl_2_, 0.5% NP-40) containing protease inhibitor cocktail (Roche, Laval, QC, Canada). Cells were then rehomogenized, incubated on ice for 5 minutes and repelleted. The pellet was resuspended in 1.5 volumes ChIP nuclear lysis buffer (1% SDS, 5 mM EDTA, 50 mM Tris-Cl, pH 8.1) supplemented with protease inhibitor cocktail tablets (Roche). Cells were passed through a 26.5 gauge needle prior to sonication in a water-ice bath (Sonicator 3000, Misonix, Farmingdale, NY, USA) for 6 cycles of 30 s on, 40 s off, and 60 μg of chromatin was pre-cleared with 250 μl Protein G beads (100 μl, Active Motif, Carlsbad, CA, USA), Protein Inhibitor Cocktail (0.5 μl, Active Motif) and supplemented with 7.5 μl ChIP dilution buffer (0.1% SDS, 10% Triton X-100, 1.67 M NaCl, 167 M Tris-Cl, pH 8.1). Beads were precipitated and 3 μg of rabbit α-Foxa2 (M-20; Santa Cruz, Santa Cruz, CA, USA), rabbit α-Pdx1 (Millipore, Billerica, MA, USA) antibody or normal rabbit IgG (Santa Cruz) was added to supernatants. Fresh Protein G beads were also blocked with 1 mg/mg bovine serum albumin and 0.1 mg/mg salmon sperm DNA in ChIP dilution buffer. Following overnight incubation, rocking at 4°C, the samples were incubated with the blocked beads for 4 h, rocking at 4°C. The beads were then precipitated and washed in low salt buffer (0.1% SDS, 1% Triton X-100, 2 mM EDTA, 20 mM Tris-Cl, pH 8.1, 150 mM NaCl), high salt buffer (low salt buffer with 500 mM NaCl), lithium chloride buffer (0.25 M LiCl, 1% NP-40, 1% deoxycholate, 1 mM EDTA, 10 mM Tris-Cl, pH 8.1) and 2× TE buffer (10 mM Trix-HCl, ph 8.0, 1 mM EDTA). Beads were resuspended in 125 μl elution buffer (1% SDS, 0.1 M NHCO_3_) and rotated for 1 h at room temperature. NaCl (0.192 M) was added to reverse crosslinks and samples were incubated overnight at 65°C. Samples were then incubated with Proteinase K (Invitrogen, Carlsbad, CA, USA) and RNaseA (Sigma, Oakville, ON, Canada) for 1 h at 50°C. DNA was purified by two rounds of phenol-chloroform extraction and ethanol precipitation and resuspended in 50 μl dH_2_O. To detect enrichment, we used qRT-PCR, as described above, using the primers listed in Additional data file 5.

### Accession numbers

The GenBank [33] accession numbers for the genes discussed are: *Pdx1 *(NM_008814), *Ptf1a *(NM_018809), *Neurog3 *(NM_009719), *Neurod1 *(NM_010894), *Pax6 *(NM_013627), *Isl1 *(NM_021459), *Nkx2-2 *(NM_010919), *Nkx6-1 *(NM_144955), *Myt3 *(NM_173868), *Foxa2 *(NM_010446), *Mafa *(NM_194350), *Pax4 *(NM_011038), *Bhlhb8 *(NM_010800), *Gcg *(NM_008100), *Iapp *(NM_010491), *Ins1 *(NM_008386), *Ins2 *(NM_008387), *Pnlip *(NM_026925), *Sdha *(NM_023281), *HbS1L *(NM_001042593), *B2m *(NM_009735), *Plunc *(NM_011126), *Cldn13 *(NM_020504), *Pomc *(NM_008895), *Prm2 *(NM_008933), *Alb *(NM_009654), *Amy1 *(NM_007446), *Gast *(NM_010257), *Tcf12 *(NM_011544), *Zfp326 *(NM_018759), *Meox1 *(NM_010791), *Zfp446 *(NM_175558), *Rnf6 *(NM_028774), *Son *(ENSMUSG00000022961), *Nr1d1 *(NM_145434), *Bcl6b *(NM_007528), *Arx *(ENSMUSG00000035277), *Mfng *(NM_008595), *Hes1 *(NM_008235), *Notch1 *(NM_008714), *β-actin *(NM_007393), *Foxa2 *(NM_010446), *Myt1 *(NM_008665), *Myt1L *(NM_001093775), *Ela3 *(NM_026419), *Pnliprp1 *(NM_018874), *Sox9 *(NM_011448), *Prrx1 *(BC092372), *Ets1 *(BC114351).

## Abbreviations

ChIP, chromatin immunoprecipitation; EGFP, enhanced green fluorescent protein; EST, expressed sequence tag; FACS, fluorescence activated cell sorting; GO, Gene Ontology; KEGG, Kyoto Encyclopedia of Genes and Genomes; PBS, phosphate buffered saline; qRT-PCR, quantitative real time PCR; S, specificity; SAGE, Serial analysis of gene expression; SOTA, self-organizing tree algorithm; TS, Theiler stage.

## Authors' contributions

BH was involved in all aspects of the project, including design, tissue collection, data analysis, qRT-PCR validations, *GenePaint *analysis, and so on. BZ assisted with qRT-PCR and *in situ *studies, and performed the *Foxa2 *ChIP experiment. JW performed tissue collection. TA performed tissue collection. MB performed the *Pdx1 *ChIP experiment. PH is a senior author. SJ is a senior author. MM is a senior author. CH is a senior author and was involved in experimental design and planning.

## Additional data files

The following additional data files are available. Additional data file 1 is a figure showing a comparison of the number and percentage of tags that map to known pancreas ESTs or transcripts as a function of minimum tag count. Additional data file 2 is an Excel spreadsheet showing the tag counts for the 2,536 tags used in our SAGE tag *K*-means cluster analysis. Additional data file 3 is a figure showing the percentage of genes identified as *Pdx1 *EGFP+, Neurog EGFP+, or Islet enriched by Gu *et al*. [22] in each of the identified *K*-means cluster groups. Additional data file 4 is a table showing the *GenePaint*-based staining classification results. Additional data file 5 is a table listing the genes and primer sequences used in qRT-PCR validation studies. Additional data file 6 is a table listing the obtained binding information for identified transcription factors.

## Supplementary Material

Additional data file 1 Number and percentage of tags that map to known pancreas ESTs or transcripts as a function of minimum tag count. Click here for file

Additional data file 2 Tag counts for the 2,536 tags used in our SAGE tag *K*-means cluster analysis. Click here for file

Additional data file 3 Percentage of genes identified as *Pdx1 *EGFP+, Neurog EGFP+, or Islet enriched by Gu *et al*. [22] in each of the identified *K*-means cluster groups. Click here for file

Additional data file 4 *GenePaint*-based staining classification results. Click here for file

Additional data file 5 Genes and primer sequences used in qRT-PCR validation studies. Click here for file

Additional data file 6 Binding information for identified transcription factors. Click here for file
