# Supplementary material for: Identification of transcripts with enriched expression in the developing and adult pancreas
Source: Genome Biol. 2008 Jun 14;9(6):R99. doi: 10.1186/gb-2008-9-6-r99 (PMC2481431; doi:10.1186/gb-2008-9-6-r99)
Supplement: Additional data file 4 — GenePaint-based staining classification results. [file gb-2008-9-6-r99-S4.doc]

| **Table S2:** Classification of Genes into the Five Expression Domains in the Developing Pancreas using the GenePaint Database | | | | | | | | | |
| --- | --- | --- | --- | --- | --- | --- | --- | --- | --- |
| Cluster | Gene symbol | GenePaint Id#^o^ | Trunk | Tip | Epithelial | Vasculature | Mesenchyme | Not detected^#^ | Undetermined^Δ^ |
| 1 | *Lhx1* | MH346 | M^ |  |  |  |  |  |  |
| 4 | *Hes6* | MH945 | M |  |  |  |  |  |  |
| 4 | *Prodh2* | MH1670 | M |  |  |  |  |  |  |
| 5 | *Cryba2* | ES253 | M |  |  |  |  |  |  |
| 5 | *Cck* | MH784 | M |  |  |  |  |  |  |
| 5 | *Hmgn3* | EG1856 | M |  |  |  |  |  |  |
| 5 | *Gadd45g* | MH557 | M |  |  |  |  |  |  |
| 5 | *AI987662* | EG318 | M |  |  |  |  |  |  |
| 5 | *Pou3f4* | MH930 | M |  |  |  |  |  |  |
| 5 | *Hap1* | MH834 | M |  |  |  |  |  |  |
| 5 | *Myt1* | EG854 | M |  |  |  |  |  |  |
| 5 | *Nkx2-2* | EN1299 | M |  |  |  |  |  |  |
| 5 | *Btg2* | EH469 | M |  |  |  |  |  |  |
| 5 | *Gpd2* | MH1609 | M |  |  |  |  |  |  |
| 5 | *Tekt2* | EB895 | M |  |  |  |  |  |  |
| 5 | *Pcsk1* | HD35 | M |  |  |  |  |  |  |
| 5 | *Abcb9* | EH2977 | M |  |  |  |  |  |  |
| 5 | *Dusp10* | MH1605 | M |  |  |  |  |  |  |
| 5 | *Ush1c* | EB2270 | M |  |  |  |  |  |  |
| 5 | *Irx2* | MY264 | M |  |  |  |  |  |  |
| 7 | *Egr1* | EN1207 | M |  |  |  |  |  |  |
| 8 | *Abcc8* | EB2685 | M |  |  |  |  |  |  |
| 8 | *Syt13* | MH494 | M |  |  |  |  |  |  |
| 8 | *Pcsk1n* | MH767 | M |  |  |  |  |  |  |
| 8 | *4930544G21Rik* | EH3424 | M |  |  |  |  |  |  |
| 8 | *Insrr* | EH3320 | M |  |  |  |  |  |  |
| 8 | *Slc7a14* | EB855 | M |  |  |  |  |  |  |
| 8 | *Mlxipl* | WB159 | M |  |  |  |  |  |  |
| 8 | *Mageh1* | HD8 | M |  |  |  |  |  |  |
| 13 | *BC038479* | EH277 | M |  |  |  |  |  |  |
| 13 | *Tnrc4* | EB2164 | M |  |  |  |  |  |  |
| 13 | *Ppp1r1a* | EG235 | M |  |  |  |  |  |  |
| 13 | *Slc2a2* | ES905 | M |  |  |  |  |  |  |
| 5 | *Gast* | EH2257 | S |  |  |  |  |  |  |
| 4 | *Sox9* | MH1164 | S |  |  |  |  |  |  |
| 4 | *Adamts1* | MY280 | S |  |  |  |  |  |  |
| 5 | *Pyy* | EH3699 | S |  |  |  |  |  |  |
| 5 | *Slc38a5* | EB330 | S |  |  |  |  |  |  |
| 5 | *Ttr* | EG1982 | S |  |  |  |  |  |  |
| 5 | *Rbp4* | EH1026 | S |  |  |  |  |  |  |
| 5 | *Ghrl* | MH779 | S |  |  |  |  |  |  |
| 5 | *Neurog3* | DA126 | S |  |  |  |  |  |  |
| 5 | *Aplp1* | EG807 | S |  |  |  |  |  |  |
| 5 | *Cdkn1a* | MH192 | S |  |  |  |  |  |  |
| 5 | *Pam* | EB1828 | S |  |  |  |  |  |  |
| 5 | *Isl1* | MH342 | S |  |  |  |  |  |  |
| 5 | *Chgb* | EH2633 | S |  |  |  |  |  |  |
| 5 | *Neurod1* | DA125 | S |  |  |  |  |  |  |
| 5 | *1700086L19Rik* | MH705 | S |  |  |  |  |  |  |
| 5 | *Entpd3* | EH2478 | S |  |  |  |  |  |  |
| 5 | *BC052055* | EG1794 | S |  |  |  |  |  |  |
| 5 | *Arx* | MH740 | S |  |  |  |  |  |  |
| 7 | *E430002G05Rik* | EH1368 | S |  |  |  |  |  |  |
| 8 | *Gcg* | EH3558 | S |  |  |  |  |  |  |
| 8 | *Scgn* | EH1443 | S |  |  |  |  |  |  |
| 8 | *Neurod1* | DA125 | S |  |  |  |  |  |  |
| 8 | *Pax6* | MH454 | S |  |  |  |  |  |  |
| 8 | *Hap1* | MH834 | S |  |  |  |  |  |  |
| 13 | *Ins2* | EB1422 | S |  |  |  |  |  |  |
| 13 | *Iapp* | EG1141 | S |  |  |  |  |  |  |
| 13 | *Pcsk2* | EH152 | S |  |  |  |  |  |  |
| 13 | *Scg3* | EH252 | S |  |  |  |  |  |  |
| 13 | *Scg2* | MH877 | S |  |  |  |  |  |  |
| 13 | *Chga* | ES454 | S |  |  |  |  |  |  |
| 13 | *Scg2* | MH877 | S |  |  |  |  |  |  |
| 13 | *Gpx3* | EN612 | S |  |  |  |  |  |  |
| 5 | *Fos* | MH982 | W |  |  |  |  |  |  |
| 4 | *Pdx1* | EN1831 | W |  |  |  |  |  |  |
| 4 | *Arg1* | EH1208 | W |  |  |  |  |  |  |
| 5 | *Grik5* | EG1805 | W |  |  |  |  |  |  |
| 5 | *Fxyd3* | EG561 | W |  |  |  |  |  |  |
| 5 | *Dnajc12* | EG143 | W |  |  |  |  |  |  |
| 5 | *Cda* | MH1652 | W |  |  |  |  |  |  |
| 5 | *Rem2* | EH1280 | W |  |  |  |  |  |  |
| 5 | *Gdap1l1* | EH3794 | W |  |  |  |  |  |  |
| 5 | *Efcab1* | EH3079 | W |  |  |  |  |  |  |
| 5 | *Pde1c* | MH905 | W |  |  |  |  |  |  |
| 8 | *Pla2g2f* | MH1625 | W |  |  |  |  |  |  |
| 8 | *Pacrg* | EB1824 | W |  |  |  |  |  |  |
| 8 | *Gck* | MH1608 | W |  |  |  |  |  |  |
| 8 | *Slc36a1* | ES1681 | W |  |  |  |  |  |  |
| 1 | *Onecut1* | EH1669 |  | M |  |  |  |  |  |
| 3 | *Fh1* | MH954 |  | M |  |  |  |  |  |
| 5 | *Cpn1* | ES1927 |  | M |  |  |  |  |  |
| 5 | *Dct* | EN1068 |  | M |  |  |  |  |  |
| 6 | *Plk3* | EH2709 |  | M |  |  |  |  |  |
| 6 | *Rpl7l1* | EH2644 |  | M |  |  |  |  |  |
| 6 | *Ide* | MH1616 |  | M |  |  |  |  |  |
| 8 | *Ddc* | MH828 |  | M |  |  |  |  |  |
| 10 | *Rap1gap* | EG1860 |  | M |  |  |  |  |  |
| 10 | *Dhx34* | EH3511 |  | M |  |  |  |  |  |
| 10 | *Id2* | EN343 |  | M |  |  |  |  |  |
| 10 | *Bhlhb8* | EB2201 |  | M |  |  |  |  |  |
| 10 | *Ppib* | EH764 |  | M |  |  |  |  |  |
| 10 | *Idh2* | MH1299 |  | M |  |  |  |  |  |
| 10 | *Hsp90aa1* | EB2411 |  | M |  |  |  |  |  |
| 10 | *Abcf2* | HD69 |  | M |  |  |  |  |  |
| 10 | *Sympk* | EH4074 |  | M |  |  |  |  |  |
| 10 | *Copg* | HD68 |  | M |  |  |  |  |  |
| 10 | *Suclg1* | MH1312 |  | M |  |  |  |  |  |
| 10 | *Rrm1* | MH1636 |  | M |  |  |  |  |  |
| 10 | *Slc25a3* | MH215 |  | M |  |  |  |  |  |
| 11 | *Spp1* | ES224 |  | M |  |  |  |  |  |
| 11 | *Mat1a* | EH952 |  | M |  |  |  |  |  |
| 13 | *Serpina10* | EH1166 |  | M |  |  |  |  |  |
| 14 | *Rpl37a* | EH2447 |  | M |  |  |  |  |  |
| 10 | *Ctrb1* | EH2594 |  | S |  |  |  |  |  |
| 10 | *Pnliprp1* | EH722 |  | S |  |  |  |  |  |
| 10 | *Spink3* | EN1013 |  | S |  |  |  |  |  |
| 10 | *Fkbp11* | EN217 |  | S |  |  |  |  |  |
| 10 | *Serpina6* | EH2400 |  | S |  |  |  |  |  |
| 10 | *Rbpjl* | EG1235 |  | S |  |  |  |  |  |
| 10 | *P2rx1* | EB486 |  | S |  |  |  |  |  |
| 10 | *Cckar* | EH3710 |  | S |  |  |  |  |  |
| 10 | *Noxa1* | ES409 |  | S |  |  |  |  |  |
| 10 | *Ak3* | EH1571 |  | S |  |  |  |  |  |
| 10 | *Nr2f6* | MH446 |  | S |  |  |  |  |  |
| 11 | *Clps* | EG997 |  | S |  |  |  |  |  |
| 11 | *Cel* | EN1129 |  | S |  |  |  |  |  |
| 11 | *Reg1* | EH2504 |  | S |  |  |  |  |  |
| 11 | *Nupr1* | ES573 |  | S |  |  |  |  |  |
| 11 | *Amy1* | ES1186 |  | S |  |  |  |  |  |
| 11 | *Xbp1* | MH1040 |  | S |  |  |  |  |  |
| 11 | *Cpa1* | ES1868 |  | S |  |  |  |  |  |
| 11 | *Cldn3* | WB115 |  | S |  |  |  |  |  |
| 12 | *Ela1* | EB2260 |  | S |  |  |  |  |  |
| 12 | *2210010C04Rik* | EH2665 |  | S |  |  |  |  |  |
| 12 | *Ctrl* | EH2595 |  | S |  |  |  |  |  |
| 12 | *Clu* | MH787 |  | S |  |  |  |  |  |
| 12 | *Ces3* | EG142 |  | S |  |  |  |  |  |
| 12 | *P2rx1* | EB486 |  | S |  |  |  |  |  |
| 14 | *Rnase4* | EH2527 |  | S |  |  |  |  |  |
| 1 | *Bex4* | ES1292 |  | W |  |  |  |  |  |
| 1 | *Hmga1* | EH1889 |  | W |  |  |  |  |  |
| 1 | *Afp* | EH1510 |  | W |  |  |  |  |  |
| 1 | *Lrig3* | ST70 |  | W |  |  |  |  |  |
| 4 | *Gata4* | EN1213 |  | W |  |  |  |  |  |
| 4 | *Nr0b2* | EH2288 |  | W |  |  |  |  |  |
| 10 | *Pgam2* | MH2059 |  | W |  |  |  |  |  |
| 10 | *Alg3* | ES473 |  | W |  |  |  |  |  |
| 10 | *Arhgdig* | EG1118 |  | W |  |  |  |  |  |
| 10 | *Surf4* | ES440 |  | W |  |  |  |  |  |
| 10 | *Edg3* | EN1828 |  | W |  |  |  |  |  |
| 10 | *Tmed6* | EN998 |  | W |  |  |  |  |  |
| 10 | *H47* | ES197 |  | W |  |  |  |  |  |
| 10 | *Acp5* | EH2024 |  | W |  |  |  |  |  |
| 10 | *Casp3* | MH1706 |  | W |  |  |  |  |  |
| 10 | *Gulo* | EN1932 |  | W |  |  |  |  |  |
| 10 | *Alg8* | EH464 |  | W |  |  |  |  |  |
| 10 | *Eif4ebp1* | ES695 |  | W |  |  |  |  |  |
| 10 | *Sdhc* | MH1427 |  | W |  |  |  |  |  |
| 10 | *Gnmt* | EH1019 |  | W |  |  |  |  |  |
| 10 | *Zdhhc4* | EH3782 |  | W |  |  |  |  |  |
| 10 | *Hspa5* | ES1203 |  | W |  |  |  |  |  |
| 10 | *Rpp14* | MH1490 |  | W |  |  |  |  |  |
| 10 | *Khsrp* | EH3322 |  | W |  |  |  |  |  |
| 10 | *Slbp* | EB2392 |  | W |  |  |  |  |  |
| 10 | *Spdef* | DA75 |  | W |  |  |  |  |  |
| 11 | *Pnliprp2* | EB550 |  | W |  |  |  |  |  |
| 11 | *Txnl1* | ES1117 |  | W |  |  |  |  |  |
| 11 | *Zdhhc2* | Zdhhc2 |  | W |  |  |  |  |  |
| 11 | *P4hb* | EH1084 |  | W |  |  |  |  |  |
| 11 | *Rnf43* | EG1315 |  | W |  |  |  |  |  |
| 12 | *Anxa13* | ES887 |  | W |  |  |  |  |  |
| 12 | *Eif2ak3* | HD27 |  | W |  |  |  |  |  |
| 12 | *Ehf* | EN1696 |  | W |  |  |  |  |  |
| 12 | *Tor3a* | EB737 |  | W |  |  |  |  |  |
| 12 | *D14Ertd668e* | EH1516 |  | W |  |  |  |  |  |
| 14 | *Gch1* | EB402 |  | W |  |  |  |  |  |
| 1 | *Akr1c13* | EH1658 |  |  | M |  |  |  |  |
| 4 | *Calm1* | MH742 |  |  | M |  |  |  |  |
| 4 | *Psma1* | EH4028 |  |  | M |  |  |  |  |
| 4 | *Tyms* | EN645 |  |  | M |  |  |  |  |
| 4 | *Nxt1* | EN462 |  |  | M |  |  |  |  |
| 4 | *Habp2* | EG1122 |  |  | M |  |  |  |  |
| 4 | *AI428936* | EB586 |  |  | M |  |  |  |  |
| 4 | *F11r* | ES800 |  |  | M |  |  |  |  |
| 4 | *Mrpl53* | EH2679 |  |  | M |  |  |  |  |
| 5 | *Vdac2* | DC33 |  |  | M |  |  |  |  |
| 5 | *Ddost* | EB1487 |  |  | M |  |  |  |  |
| 5 | *Tmed3* | EH1624 |  |  | M |  |  |  |  |
| 5 | *Cited1* | EG39 |  |  | M |  |  |  |  |
| 5 | *Mrps18c* | ES286 |  |  | M |  |  |  |  |
| 5 | *Garnl4* | EN1348 |  |  | M |  |  |  |  |
| 5 | *Stxbp2* | ES737 |  |  | M |  |  |  |  |
| 5 | *Ffar2* | EH3636 |  |  | M |  |  |  |  |
| 5 | *Elf3* | EN322 |  |  | M |  |  |  |  |
| 5 | *Ssr1* | EH3589 |  |  | M |  |  |  |  |
| 5 | *Gstz1* | EN886 |  |  | M |  |  |  |  |
| 5 | *Kirrel2* | EN803 |  |  | M |  |  |  |  |
| 5 | *Cnot6l* | EH2029 |  |  | M |  |  |  |  |
| 5 | *Rassf6* | EH1927 |  |  | M |  |  |  |  |
| 5 | *Sdcbp2* | EH2136 |  |  | M |  |  |  |  |
| 5 | *Gpx2* | ES407 |  |  | M |  |  |  |  |
| 5 | *9130017N09Rik* | EH1219 |  |  | M |  |  |  |  |
| 5 | *Akap9* | EG11 |  |  | M |  |  |  |  |
| 5 | *Faah* | EH1211 |  |  | M |  |  |  |  |
| 5 | *Foxa3* | EH531 |  |  | M |  |  |  |  |
| 5 | *Tnk1* | EH3040 |  |  | M |  |  |  |  |
| 5 | *Bex2* | EB2352 |  |  | M |  |  |  |  |
| 5 | *1810073N04Rik* | EG606 |  |  | M |  |  |  |  |
| 6 | *Rpl17* | ES369 |  |  | M |  |  |  |  |
| 6 | *St14* | EG1103 |  |  | M |  |  |  |  |
| 6 | *Rab3d* | EB915 |  |  | M |  |  |  |  |
| 7 | *Hpn* | EG2296 |  |  | M |  |  |  |  |
| 10 | *Mcfd2* | EH812 |  |  | M |  |  |  |  |
| 10 | *Calm2* | DC46 |  |  | M |  |  |  |  |
| 10 | *Ptdss1* | EH1861 |  |  | M |  |  |  |  |
| 10 | *Fgd4* | ES121 |  |  | M |  |  |  |  |
| 10 | *Hmga2* | EN837 |  |  | M |  |  |  |  |
| 10 | *Pofut2* | MY306 |  |  | M |  |  |  |  |
| 11 | *Atp2a2* | EB1887 |  |  | M |  |  |  |  |
| 11 | *Cd2ap* | MH1789 |  |  | M |  |  |  |  |
| 11 | *Zfp473* | EH2649 |  |  | M |  |  |  |  |
| 12 | *Card10* | EH3382 |  |  | M |  |  |  |  |
| 12 | *Cxadr* | ES1928 |  |  | M |  |  |  |  |
| 13 | *Acly* | EH152 |  |  | M |  |  |  |  |
| 13 | *Lgals3bp* | EH1258 |  |  | M |  |  |  |  |
| 14 | *Rps11* | EH2266 |  |  | M |  |  |  |  |
| 14 | *Rps7* | EH1848 |  |  | M |  |  |  |  |
| 14 | *Rpl14* | EH1586 |  |  | M |  |  |  |  |
| 14 | *Mdk* | MH609 |  |  | M |  |  |  |  |
| 14 | *Maged1* | ES1941 |  |  | M |  |  |  |  |
| 14 | *Pdia6* | EN640 |  |  | M |  |  |  |  |
| 14 | *Psma7* | EH939 |  |  | M |  |  |  |  |
| 14 | *Foxa2* | MH515 |  |  | M |  |  |  |  |
| 14 | *Naca* | EB196 |  |  | M |  |  |  |  |
| 4 | *Ambp* | EH2278 |  |  | S |  |  |  |  |
| 4 | *Foxa2* | MH515 |  |  | S |  |  |  |  |
| 5 | *Spint2* | EG2099 |  |  | S |  |  |  |  |
| 5 | *Serpina1a* | EG416 |  |  | S |  |  |  |  |
| 5 | *Emb* | EH1517 |  |  | S |  |  |  |  |
| 5 | *Tle6* | MH1997 |  |  | S |  |  |  |  |
| 5 | *Anxa4* | EH789 |  |  | S |  |  |  |  |
| 5 | *Sox9* | MH1164 |  |  | S |  |  |  |  |
| 5 | *Serpina1b* | EG453 |  |  | S |  |  |  |  |
| 5 | *Adamts16* | EN411 |  |  | S |  |  |  |  |
| 6 | *Tacstd1* | ST62 |  |  | S |  |  |  |  |
| 6 | *Gc* | EH1017 |  |  | S |  |  |  |  |
| 6 | *Aldh1b1* | EH3923 |  |  | S |  |  |  |  |
| 6 | *4922503N01Rik* | EH357 |  |  | S |  |  |  |  |
| 7 | *Txnip* | DC32 |  |  | S |  |  |  |  |
| 7 | *Cdh1* | DA93 |  |  | S |  |  |  |  |
| 8 | *Anpep* | ES690 |  |  | S |  |  |  |  |
| 12 | *1700011H14Rik* | EH1371 |  |  | S |  |  |  |  |
| 12 | *Cgn* | EB1895 |  |  | S |  |  |  |  |
| 12 | *Kcnq1* | ES528 |  |  | S |  |  |  |  |
| 14 | *Krt18* | EH3297 |  |  | S |  |  |  |  |
| 14 | *Dap* | EH128 |  |  | S |  |  |  |  |
| 14 | *Pcbd1* | EN540 |  |  | S |  |  |  |  |
| 1 | *5830467P10Rik* | ES1974 |  |  | W |  |  |  |  |
| 1 | *Coq7* | EH1040 |  |  | W |  |  |  |  |
| 1 | *Pdlim1* | EH1085 |  |  | W |  |  |  |  |
| 1 | *Fkbp4* | EB1162 |  |  | W |  |  |  |  |
| 1 | *Cenph* | EH1821 |  |  | W |  |  |  |  |
| 2 | *Lsm6* | EH3906 |  |  | W |  |  |  |  |
| 2 | *Maml3* | EB1234 |  |  | W |  |  |  |  |
| 2 | *Actl6a* | EH3945 |  |  | W |  |  |  |  |
| 2 | *Zfp191* | EH3736 |  |  | W |  |  |  |  |
| 2 | *Zfp326* | EH210 |  |  | W |  |  |  |  |
| 2 | *Cdc2l1* | ES1161 |  |  | W |  |  |  |  |
| 4 | *Eif5a* | EN1739 |  |  | W |  |  |  |  |
| 4 | *Lars2* | EH3300 |  |  | W |  |  |  |  |
| 4 | *Uqcrfs1* | EG455 |  |  | W |  |  |  |  |
| 4 | *D19Ertd721e* | EH1133 |  |  | W |  |  |  |  |
| 4 | *Psmc1* | EG2239 |  |  | W |  |  |  |  |
| 4 | *H2-Ke2* | ES387 |  |  | W |  |  |  |  |
| 4 | *Srrm1* | EN1418 |  |  | W |  |  |  |  |
| 4 | *Diablo* | ES797 |  |  | W |  |  |  |  |
| 4 | *Mrpl4* | EH1277 |  |  | W |  |  |  |  |
| 4 | *Nol5* | EB1317 |  |  | W |  |  |  |  |
| 4 | *Ap1g2* | EB1879 |  |  | W |  |  |  |  |
| 4 | *Mov10* | ES1895 |  |  | W |  |  |  |  |
| 4 | *1700020C11Rik* | EN516 |  |  | W |  |  |  |  |
| 4 | *Cldn9* | EB564 |  |  | W |  |  |  |  |
| 4 | *Rreb1* | EB1711 |  |  | W |  |  |  |  |
| 4 | *Adfp* | EH949 |  |  | W |  |  |  |  |
| 4 | *Papola* | MH1009 |  |  | W |  |  |  |  |
| 4 | *Wdr75* | ES1358 |  |  | W |  |  |  |  |
| 4 | *BC003331* | EH844 |  |  | W |  |  |  |  |
| 4 | *Tcerg1* | EG1811 |  |  | W |  |  |  |  |
| 4 | *Chtf18* | EG1054 |  |  | W |  |  |  |  |
| 4 | *Dad1* | EN1897 |  |  | W |  |  |  |  |
| 4 | *Asxl2* | ES194 |  |  | W |  |  |  |  |
| 5 | *Ssr2* | EB312 |  |  | W |  |  |  |  |
| 5 | *2310003F16Rik* | EH1127 |  |  | W |  |  |  |  |
| 5 | *Ndufb5* | EG714 |  |  | W |  |  |  |  |
| 5 | *2900010J23Rik* | ES297 |  |  | W |  |  |  |  |
| 5 | *D11Bwg0434e* | EH1515 |  |  | W |  |  |  |  |
| 5 | *Cdc26* | EH1898 |  |  | W |  |  |  |  |
| 5 | *Mrps18a* | EG43 |  |  | W |  |  |  |  |
| 5 | *Peg3* | EN1335 |  |  | W |  |  |  |  |
| 5 | *Erp29* | EG269 |  |  | W |  |  |  |  |
| 5 | *5730469M10Rik* | EN544 |  |  | W |  |  |  |  |
| 5 | *0610011F06Rik* | EH2403 |  |  | W |  |  |  |  |
| 5 | *1810047C23Rik* | ES588 |  |  | W |  |  |  |  |
| 5 | *Stk16* | ES744 |  |  | W |  |  |  |  |
| 5 | *Ppp6c* | EH3567 |  |  | W |  |  |  |  |
| 5 | *2010107G23Rik* | ES2001 |  |  | W |  |  |  |  |
| 5 | *Gnb2* | MH634 |  |  | W |  |  |  |  |
| 5 | *Serpinf2* | EH1055 |  |  | W |  |  |  |  |
| 5 | *2310030G06Rik* | EH2897 |  |  | W |  |  |  |  |
| 5 | *4833418A01Rik* | EH1892 |  |  | W |  |  |  |  |
| 5 | *Akr1c12* | EG1887 |  |  | W |  |  |  |  |
| 5 | *Capsl* | EH2771 |  |  | W |  |  |  |  |
| 5 | *Lman1* | EN287 |  |  | W |  |  |  |  |
| 5 | *Zfp707* | EB1715 |  |  | W |  |  |  |  |
| 5 | *1200016B10Rik* | EH2650 |  |  | W |  |  |  |  |
| 5 | *Fuca1* | ES42 |  |  | W |  |  |  |  |
| 5 | *Dicer1* | MH2221 |  |  | W |  |  |  |  |
| 5 | *Jtb* | EG174 |  |  | W |  |  |  |  |
| 5 | *St6galnac2* | EH2066 |  |  | W |  |  |  |  |
| 5 | *Nup54* | ES976 |  |  | W |  |  |  |  |
| 5 | *Pitpnb* | ES1942 |  |  | W |  |  |  |  |
| 5 | *Gyltl1b* | EH2394 |  |  | W |  |  |  |  |
| 5 | *Enpp4* | EH1482 |  |  | W |  |  |  |  |
| 5 | *Tspan15* | EH1066 |  |  | W |  |  |  |  |
| 5 | *Rai2* | EH2827 |  |  | W |  |  |  |  |
| 5 | *Hist1h1d* | EH758 |  |  | W |  |  |  |  |
| 5 | *3110032G18Rik* | EG570 |  |  | W |  |  |  |  |
| 5 | *BC050092* | ES793 |  |  | W |  |  |  |  |
| 5 | *Tbk1* | MH1494 |  |  | W |  |  |  |  |
| 5 | *2810002I04Rik* | EH626 |  |  | W |  |  |  |  |
| 5 | *Bcap29* | EH1149 |  |  | W |  |  |  |  |
| 5 | *4632417K18Rik* | EH3887 |  |  | W |  |  |  |  |
| 5 | *Orc4l* | EH198 |  |  | W |  |  |  |  |
| 5 | *2810451A06Rik* | EG440 |  |  | W |  |  |  |  |
| 5 | *Zfp113* | EN54 |  |  | W |  |  |  |  |
| 5 | *Taf15* | EN1445 |  |  | W |  |  |  |  |
| 5 | *2310008M10Rik* | EG421 |  |  | W |  |  |  |  |
| 5 | *Mycbpap* | EN741 |  |  | W |  |  |  |  |
| 5 | *Tspan6* | ES711 |  |  | W |  |  |  |  |
| 5 | *Mrps31* | EH3804 |  |  | W |  |  |  |  |
| 5 | *Ccdc16* | ES353 |  |  | W |  |  |  |  |
| 6 | *Cnih* | MH153 |  |  | W |  |  |  |  |
| 6 | *Traf7* | EH1744 |  |  | W |  |  |  |  |
| 6 | *Chd3* | EH3842 |  |  | W |  |  |  |  |
| 6 | *Ufm1* | ES2325 |  |  | W |  |  |  |  |
| 7 | *3110049J23Rik* | EH2531 |  |  | W |  |  |  |  |
| 8 | *Rpn2* | EH2506 |  |  | W |  |  |  |  |
| 8 | *Tcf25* | EH3754 |  |  | W |  |  |  |  |
| 8 | *Adrbk2* | EB1753 |  |  | W |  |  |  |  |
| 8 | *Cyp4f13* | ES562 |  |  | W |  |  |  |  |
| 8 | *BC026682* | ES393 |  |  | W |  |  |  |  |
| 8 | *Slc16a12* | EG1332 |  |  | W |  |  |  |  |
| 9 | *Rps27* | EG873 |  |  | W |  |  |  |  |
| 9 | *Rps3* | EN512 |  |  | W |  |  |  |  |
| 10 | *Actc1* | DC37 |  |  | W |  |  |  |  |
| 10 | *Gcnt1* | EH368 |  |  | W |  |  |  |  |
| 10 | *Derl1* | EH3482 |  |  | W |  |  |  |  |
| 10 | *Ucp2* | EG1912 |  |  | W |  |  |  |  |
| 10 | *Lgi2* | EH3345 |  |  | W |  |  |  |  |
| 10 | *Nedd4* | EB2705 |  |  | W |  |  |  |  |
| 10 | *Nsmce1* | EN914 |  |  | W |  |  |  |  |
| 10 | *Ppp4r1* | EH1379 |  |  | W |  |  |  |  |
| 10 | *Cchcr1* | ES1977 |  |  | W |  |  |  |  |
| 10 | *Smurf1* | EH1999 |  |  | W |  |  |  |  |
| 10 | *Sc5d* | EH1701 |  |  | W |  |  |  |  |
| 10 | *Azin1* | EH3988 |  |  | W |  |  |  |  |
| 10 | *Mga* | EB361 |  |  | W |  |  |  |  |
| 10 | *Tcf7l2* | HD19 |  |  | W |  |  |  |  |
| 10 | *Acadl* | ES885 |  |  | W |  |  |  |  |
| 10 | *Slc31a1* | ES776 |  |  | W |  |  |  |  |
| 10 | *Gstt1* | MH1270 |  |  | W |  |  |  |  |
| 11 | *Cyp4v3* | EH3133 |  |  | W |  |  |  |  |
| 11 | *Cblc* | ES627 |  |  | W |  |  |  |  |
| 12 | *Csrp1* | HD13 |  |  | W |  |  |  |  |
| 12 | *Kifc3* | EH3238 |  |  | W |  |  |  |  |
| 12 | *Ly6e* | EG2048 |  |  | W |  |  |  |  |
| 12 | *Hhex* | EB22 |  |  | W |  |  |  |  |
| 12 | *Tjp3* | EH2100 |  |  | W |  |  |  |  |
| 12 | *9030611O19Rik* | EH1218 |  |  | W |  |  |  |  |
| 12 | *Itga3* | MH406 |  |  | W |  |  |  |  |
| 12 | *Kif1c* | EH3280 |  |  | W |  |  |  |  |
| 13 | *Rgs11* | EH1119 |  |  | W |  |  |  |  |
| 13 | *0610007L01Rik* | ES2127 |  |  | W |  |  |  |  |
| 13 | *Lrrn1* | EH491 |  |  | W |  |  |  |  |
| 13 | *Kcnj11* | EB1640 |  |  | W |  |  |  |  |
| 13 | *Dnajc6* | ES1331 |  |  | W |  |  |  |  |
| 14 | *Itm2b* | EB891 |  |  | W |  |  |  |  |
| 14 | *0610038D11Rik* | EG1974 |  |  | W |  |  |  |  |
| 1 | *Ube2c* | EN1700 |  |  | W |  |  |  |  |
| 1 | *Prpf4b* | EG1080 |  |  | W |  |  |  |  |
| 1 | *2310044H10Rik* | EG751 |  |  | W |  |  |  |  |
| 1 | *Klf5* | EH581 |  |  | W |  |  |  |  |
| 1 | *Usp46* | EN422 |  |  | W |  |  |  |  |
| 4 | *6330577E15Rik* | ES449 |  |  | W |  |  |  |  |
| 5 | *Son* | MH1010 |  |  | W |  |  |  |  |
| 14 | *Acta2* | EH2333 |  |  | W |  |  |  |  |
| 2 | *D10Wsu102e* | EB1355 |  |  | W |  |  |  |  |
| 1 | *Cxcl4* | ES2254 |  |  |  | M |  |  |  |
| 4 | *Trf* | EH987 |  |  |  | M |  |  |  |
| 5 | *Ctsf* | EH215 |  |  |  | M |  |  |  |
| 5 | *Sct* | ES67 |  |  |  | M |  |  |  |
| 5 | *AI836003* | EH2533 |  |  |  | M |  |  |  |
| 5 | *Golga4* | MH681 |  |  |  | M |  |  |  |
| 9 | *Lgals1* | EG486 |  |  |  | M |  |  |  |
| 9 | *Mest* | EH1877 |  |  |  | M |  |  |  |
| 10 | *Myl9* | EB2526 |  |  |  | M |  |  |  |
| 10 | *Tnnc1* | EH1249 |  |  |  | M |  |  |  |
| 10 | *Ugt2b34* | EH1536 |  |  |  | M |  |  |  |
| 10 | *Ass1* | MH1600 |  |  |  | M |  |  |  |
| 10 | *Tinagl* | EN1113 |  |  |  | M |  |  |  |
| 12 | *Ltbp4* | EG56 |  |  |  | M |  |  |  |
| 12 | *Anxa3* | EN974 |  |  |  | M |  |  |  |
| 12 | *Gas6* | EH1238 |  |  |  | M |  |  |  |
| 12 | *Ctgf* | EH1110 |  |  |  | M |  |  |  |
| 12 | *Kcne3* | EH3746 |  |  |  | M |  |  |  |
| 12 | *Cpxm2* | EH150 |  |  |  | M |  |  |  |
| 13 | *Bace2* | MY255 |  |  |  | M |  |  |  |
| 13 | *Ace2* | ES886 |  |  |  | M |  |  |  |
| 10 | *Hba-a1* | EB88 |  |  |  | S |  |  |  |
| 10 | *Hbb-y* | EG744 |  |  |  | S |  |  |  |
| 10 | *Centd3* | EH3169 |  |  |  | S |  |  |  |
| 10 | *Slc4a1* | MH2262 |  |  |  | S |  |  |  |
| 12 | *Aqp1* | MH2267 |  |  |  | S |  |  |  |
| 12 | *Cfh* | EH2983 |  |  |  | S |  |  |  |
| 2 | *Cyr61* | MH1099 |  |  |  | W |  |  |  |
| 5 | *Fkbp1a* | EH1909 |  |  |  | W |  |  |  |
| 5 | *Eif4e3* | EG204 |  |  |  | W |  |  |  |
| 7 | *Fos* | MH982 |  |  |  | W |  |  |  |
| 9 | *Emp3* | EN314 |  |  |  | W |  |  |  |
| 10 | *Myl7* | EH2599 |  |  |  | W |  |  |  |
| 10 | *Ankrd1* | EH2726 |  |  |  | W |  |  |  |
| 10 | *Myl3* | EH3513 |  |  |  | W |  |  |  |
| 10 | *Cotl1* | EH3217 |  |  |  | W |  |  |  |
| 10 | *Ubr2* | EH3816 |  |  |  | W |  |  |  |
| 10 | *D730040F13Rik* | EH405 |  |  |  | W |  |  |  |
| 10 | *Map3k11* | EB171 |  |  |  | W |  |  |  |
| 10 | *Tln1* | EN1555 |  |  |  | W |  |  |  |
| 12 | *Dusp1* | MH1451 |  |  |  | W |  |  |  |
| 12 | *Eltd1* | ES306 |  |  |  | W |  |  |  |
| 12 | *Lrg1* | EG370 |  |  |  |  | M |  |  |
| 2 | *Prrx1* | DA120 |  |  |  |  | M |  |  |
| 3 | *Mfap2* | EH1431 |  |  |  |  | M |  |  |
| 3 | *Rgs5* | EB1449 |  |  |  |  | M |  |  |
| 5 | *Serping1* | EH1004 |  |  |  |  | M |  |  |
| 9 | *Cdkn1c* | MH1446 |  |  |  |  | M |  |  |
| 9 | *Nkx2-3* | EB1495 |  |  |  |  | M |  |  |
| 10 | *Tpm1* | EB1920 |  |  |  |  | M |  |  |
| 10 | *Nfib* | EN1771 |  |  |  |  | M |  |  |
| 10 | *Meis1* | EG1701 |  |  |  |  | M |  |  |
| 11 | *Fbln2* | EN1718 |  |  |  |  | M |  |  |
| 12 | *Sparcl1* | EN1803 |  |  |  |  | M |  |  |
| 12 | *Alcam* | EH1895 |  |  |  |  | M |  |  |
| 14 | *Tmsb4x* | EH1986 |  |  |  |  | M |  |  |
| 14 | *S100a11* | EB202 |  |  |  |  | M |  |  |
| 3 | *Sfrp1* | MH555 |  |  |  |  | S |  |  |
| 3 | *Syt6* | MH497 |  |  |  |  | S |  |  |
| 3 | *Akap12* | EB1789 |  |  |  |  | S |  |  |
| 10 | *Igfbp5* | EN601 |  |  |  |  | S |  |  |
| 10 | *Col5a2* | ES195 |  |  |  |  | S |  |  |
| 10 | *Gas1* | MH360 |  |  |  |  | S |  |  |
| 10 | *Ets1* | DA108 |  |  |  |  | S |  |  |
| 10 | *Col14a1* | EN369 |  |  |  |  | S |  |  |
| 11 | *Col3a1* | EB1311 |  |  |  |  | S |  |  |
| 12 | *Col1a1* | ES275 |  |  |  |  | S |  |  |
| 12 | *Col6a1* | MY283 |  |  |  |  | S |  |  |
| 12 | *Bgn* | ES916 |  |  |  |  | S |  |  |
| 12 | *Col15a1* | EH1224 |  |  |  |  | S |  |  |
| 12 | *Cd34* | EH921 |  |  |  |  | S |  |  |
| 12 | *Col1a2* | EB1096 |  |  |  |  | S |  |  |
| 12 | *Timp3* | EH1096 |  |  |  |  | S |  |  |
| 1 | *Nola2* | ES571 |  |  |  |  | W |  |  |
| 1 | *Fbn2* | EG1493 |  |  |  |  | W |  |  |
| 1 | *Itih2* | ES1937 |  |  |  |  | W |  |  |
| 2 | *Gypc* | EH1135 |  |  |  |  | W |  |  |
| 3 | *Emilin1* | EH1797 |  |  |  |  | W |  |  |
| 3 | *Olfml3* | EH895 |  |  |  |  | W |  |  |
| 3 | *2610027C15Rik* | ES2189 |  |  |  |  | W |  |  |
| 3 | *Nid1* | EB2054 |  |  |  |  | W |  |  |
| 3 | *Loxl2* | ES601 |  |  |  |  | W |  |  |
| 5 | *Arc* | EH2695 |  |  |  |  | W |  |  |
| 9 | *Igf2* | MH523 |  |  |  |  | W |  |  |
| 10 | *Ogn* | EH1323 |  |  |  |  | W |  |  |
| 10 | *Gsn* | MH1230 |  |  |  |  | W |  |  |
| 11 | *Klf4* | EB1445 |  |  |  |  | W |  |  |
| 12 | *Eln* | EB812 |  |  |  |  | W |  |  |
| 12 | *Tgm2* | EN1468 |  |  |  |  | W |  |  |
| 12 | *Tagln* | EB415 |  |  |  |  | W |  |  |
| 12 | *Dcn* | MH1185 |  |  |  |  | W |  |  |
| 12 | *Scara3* | EG644 |  |  |  |  | W |  |  |
| 14 | *Rcn3* | ES2158 |  |  |  |  | W |  |  |
| 10 | *Nrtn* | MH1007 |  |  |  |  |  | Y |  |
| 1 | *Crabp2* | ES629 |  |  |  |  |  | Y |  |
| 1 | *Sfrp5* | MH917 |  |  |  |  |  | Y |  |
| 1 | *Onecut2* | EN1302 |  |  |  |  |  | Y |  |
| 1 | *Rbm27* | EH4068 |  |  |  |  |  | Y |  |
| 1 | *Apoa2* | MH605 |  |  |  |  |  | Y |  |
| 1 | *Hoxb5* | EH612 |  |  |  |  |  | Y |  |
| 1 | *Foxa1* | MH864 |  |  |  |  |  | Y |  |
| 1 | *Cpm* | ES1761 |  |  |  |  |  | Y |  |
| 1 | *Paics* | ES977 |  |  |  |  |  | Y |  |
| 1 | *Neurog1* | DA72 |  |  |  |  |  | Y |  |
| 1 | *Mvk* | MH1274 |  |  |  |  |  | Y |  |
| 1 | *D14Ertd581e* | EH1225 |  |  |  |  |  | Y |  |
| 1 | *Neurod4* | DA87 |  |  |  |  |  | Y |  |
| 1 | *Hoxb1* | EN1223 |  |  |  |  |  | Y |  |
| 1 | *Pax3* | ES1828 |  |  |  |  |  | Y |  |
| 2 | *Rbp1* | EH1743 |  |  |  |  |  | Y |  |
| 2 | *Apoa1* | EH969 |  |  |  |  |  | Y |  |
| 2 | *Hoxb6* | EH1240 |  |  |  |  |  | Y |  |
| 2 | *Fusip1* | EH218 |  |  |  |  |  | Y |  |
| 2 | *Pycard* | EH110 |  |  |  |  |  | Y |  |
| 3 | *Tcf21* | EB2501 |  |  |  |  |  | Y |  |
| 3 | *Cpz* | MH1603 |  |  |  |  |  | Y |  |
| 3 | *St6galnac3* | EN1779 |  |  |  |  |  | Y |  |
| 4 | *Chd7* | EG2087 |  |  |  |  |  | Y |  |
| 4 | *Baz2b* | EH3925 |  |  |  |  |  | Y |  |
| 4 | *Meox1* | EH450 |  |  |  |  |  | Y |  |
| 5 | *Mtch1* | EG57 |  |  |  |  |  | Y |  |
| 5 | *Psmd8* | ES1070 |  |  |  |  |  | Y |  |
| 5 | *Dpf2* | MH843 |  |  |  |  |  | Y |  |
| 5 | *Mgst3* | ES486 |  |  |  |  |  | Y |  |
| 5 | *Vti1b* | EB2195 |  |  |  |  |  | Y |  |
| 5 | *Apom* | EH1696 |  |  |  |  |  | Y |  |
| 5 | *Commd9* | EN1840 |  |  |  |  |  | Y |  |
| 5 | *2810405K02Rik* | ES500 |  |  |  |  |  | Y |  |
| 5 | *Ttc3* | MH385 |  |  |  |  |  | Y |  |
| 5 | *BC034069* | EN1942 |  |  |  |  |  | Y |  |
| 5 | *Fgb* | EG2092 |  |  |  |  |  | Y |  |
| 5 | *Gal* | EG539 |  |  |  |  |  | Y |  |
| 5 | *Spink4* | EB412 |  |  |  |  |  | Y |  |
| 5 | *2310028N02Rik* | EH550 |  |  |  |  |  | Y |  |
| 5 | *Slit1* | MH2224 |  |  |  |  |  | Y |  |
| 5 | *Spp2* | EG88 |  |  |  |  |  | Y |  |
| 5 | *Plac1* | ES835 |  |  |  |  |  | Y |  |
| 5 | *Tff3* | MY224 |  |  |  |  |  | Y |  |
| 5 | *Ppp1r9a* | ES1806 |  |  |  |  |  | Y |  |
| 5 | *Asb4* | MH714 |  |  |  |  |  | Y |  |
| 5 | *Gm166* | EN1842 |  |  |  |  |  | Y |  |
| 5 | *Igfbp1* | EN838 |  |  |  |  |  | Y |  |
| 5 | *Efcab2* | EB1073 |  |  |  |  |  | Y |  |
| 5 | *Cpb2* | MH1450 |  |  |  |  |  | Y |  |
| 5 | *Suox* | MH1493 |  |  |  |  |  | Y |  |
| 5 | *Preb* | EG329 |  |  |  |  |  | Y |  |
| 5 | *1700001C02Rik* | EB708 |  |  |  |  |  | Y |  |
| 5 | *Calca* | EH905 |  |  |  |  |  | Y |  |
| 5 | *Odf2l* | EN1132 |  |  |  |  |  | Y |  |
| 5 | *Fgl1* | EH290 |  |  |  |  |  | Y |  |
| 5 | *Marveld3* | EG448 |  |  |  |  |  | Y |  |
| 5 | *Aacs* | ES884 |  |  |  |  |  | Y |  |
| 5 | *Lrrc46* | EH1390 |  |  |  |  |  | Y |  |
| 5 | *Gpatch1* | EN1373 |  |  |  |  |  | Y |  |
| 5 | *Sv2c* | MH400 |  |  |  |  |  | Y |  |
| 5 | *Dscr3* | MH389 |  |  |  |  |  | Y |  |
| 5 | *Mc4r* | MH409 |  |  |  |  |  | Y |  |
| 5 | *Slc26a2* | EH66 |  |  |  |  |  | Y |  |
| 5 | *Slc14a2* | EH726 |  |  |  |  |  | Y |  |
| 5 | *Prkce* | EB2069 |  |  |  |  |  | Y |  |
| 6 | *Col9a2* | EH749 |  |  |  |  |  | Y |  |
| 6 | *Kcnmb1* | EN1749 |  |  |  |  |  | Y |  |
| 8 | *Cadps* | ES1294 |  |  |  |  |  | Y |  |
| 8 | *St18* | EG1316 |  |  |  |  |  | Y |  |
| 8 | *Hgfac* | EG408 |  |  |  |  |  | Y |  |
| 8 | *Ubxd3* | EN2013 |  |  |  |  |  | Y |  |
| 9 | *H2afv* | EG2033 |  |  |  |  |  | Y |  |
| 9 | *Cald1* | EG423 |  |  |  |  |  | Y |  |
| 10 | *Tnni3* | DC25 |  |  |  |  |  | Y |  |
| 10 | *Myl1* | ES1562 |  |  |  |  |  | Y |  |
| 10 | *Tnni1* | EH1052 |  |  |  |  |  | Y |  |
| 10 | *Tnnt2* | EH505 |  |  |  |  |  | Y |  |
| 10 | *Csrp3* | EH3219 |  |  |  |  |  | Y |  |
| 10 | *Nppa* | EN966 |  |  |  |  |  | Y |  |
| 10 | *D0H4S114* | EG1108 |  |  |  |  |  | Y |  |
| 10 | *Cox6a2* | EG779 |  |  |  |  |  | Y |  |
| 10 | *Mybpc3* | DC40 |  |  |  |  |  | Y |  |
| 10 | *Actn2* | MH1166 |  |  |  |  |  | Y |  |
| 10 | *Emilin2* | EG674 |  |  |  |  |  | Y |  |
| 10 | *Smpx* | DC27 |  |  |  |  |  | Y |  |
| 10 | *Ldb3* | DC30 |  |  |  |  |  | Y |  |
| 10 | *Myom1* | DC35 |  |  |  |  |  | Y |  |
| 10 | *B230120H23Rik* | EB588 |  |  |  |  |  | Y |  |
| 10 | *Plxnb2* | ST51 |  |  |  |  |  | Y |  |
| 10 | *Tmod1* | EN1564 |  |  |  |  |  | Y |  |
| 10 | *Asb2* | ES267 |  |  |  |  |  | Y |  |
| 10 | *Acsl1* | MH750 |  |  |  |  |  | Y |  |
| 10 | *Plekhc1* | ES2177 |  |  |  |  |  | Y |  |
| 10 | *Gnai2* | MH625 |  |  |  |  |  | Y |  |
| 10 | *Nppb* | EH1361 |  |  |  |  |  | Y |  |
| 10 | *Myoz2* | EH1048 |  |  |  |  |  | Y |  |
| 10 | *C130038G02Rik* | EH279 |  |  |  |  |  | Y |  |
| 10 | *Dnajc5* | ES314 |  |  |  |  |  | Y |  |
| 10 | *Btk* | ES859 |  |  |  |  |  | Y |  |
| 10 | *Chrna1* | EH798 |  |  |  |  |  | Y |  |
| 12 | *H2-Aa* | EH1915 |  |  |  |  |  | Y |  |
| 12 | *Tff2* | MY222 |  |  |  |  |  | Y |  |
| 12 | *Lbp* | EG114 |  |  |  |  |  | Y |  |
| 12 | *Glipr1* | EG866 |  |  |  |  |  | Y |  |
| 12 | *Vip* | MH886 |  |  |  |  |  | Y |  |
| 12 | *Darc* | EN1745 |  |  |  |  |  | Y |  |
| 12 | *Igfbp6* | EG1943 |  |  |  |  |  | Y |  |
| 12 | *Cd44* | EN1659 |  |  |  |  |  | Y |  |
| 12 | *Adcy7* | MH1594 |  |  |  |  |  | Y |  |
| 12 | *Ace* | EH2087 |  |  |  |  |  | Y |  |
| 13 | *Tmem66* | EH3780 |  |  |  |  |  | Y |  |
| 13 | *Sytl4* | EG1790 |  |  |  |  |  | Y |  |
| 13 | *Slc7a8* | EH225 |  |  |  |  |  | Y |  |
| 13 | *Klf9* | EN1832 |  |  |  |  |  | Y |  |
| 13 | *Dhx40* | EN598 |  |  |  |  |  | Y |  |
| 13 | *Adamts15* | EB1749 |  |  |  |  |  | Y |  |
| 13 | *Frzb* | MH678 |  |  |  |  |  | Y |  |
| 13 | *Car10* | MH2174 |  |  |  |  |  | Y |  |
| 14 | *Rpl8* | ES1211 |  |  |  |  |  | Y |  |
| 1 | *Ebf2* | EN785 |  |  |  |  |  | Y |  |
| 1 | *Cdk5rap2* | ES2265 |  |  |  |  |  | Y |  |
| 1 | *Dcc* | EH3202 |  |  |  |  |  | Y |  |
| 5 | *5830434P21Rik* | ES1796 |  |  |  |  |  |  | Y |
| 1 | *Tdh* | ES1950 |  |  |  |  |  |  | Y |
| 1 | *Kctd14* | ES2173 |  |  |  |  |  |  | Y |
| 1 | *1190003M12Rik* | EG1612 |  |  |  |  |  |  | Y |
| 1 | *Dock7* | EH3953 |  |  |  |  |  |  | Y |
| 1 | *Chka* | ES1818 |  |  |  |  |  |  | Y |
| 1 | *Rad50* | EG86 |  |  |  |  |  |  | Y |
| 1 | *Nf1* | ES1724 |  |  |  |  |  |  | Y |
| 1 | *Qser1* | EG1220 |  |  |  |  |  |  | Y |
| 1 | *Krt20* | EB1446 |  |  |  |  |  |  | Y |
| 1 | *Epc1* | ES2414 |  |  |  |  |  |  | Y |
| 1 | *Fsd1l* | EH3143 |  |  |  |  |  |  | Y |
| 1 | *Ubp1* | EN293 |  |  |  |  |  |  | Y |
| 1 | *Psmc6* | EB1948 |  |  |  |  |  |  | Y |
| 1 | *Dio3* | EN1446 |  |  |  |  |  |  | Y |
| 1 | *Pard3b* | ES1606 |  |  |  |  |  |  | Y |
| 2 | *C430003P19Rik* | EN197 |  |  |  |  |  |  | Y |
| 2 | *Narg1l* | EG1143 |  |  |  |  |  |  | Y |
| 4 | *Gemin7* | EH2481 |  |  |  |  |  |  | Y |
| 4 | *1810063B05Rik* | EG820 |  |  |  |  |  |  | Y |
| 4 | *Samm50* | EB1712 |  |  |  |  |  |  | Y |
| 4 | *Fbxo16* | EG1504 |  |  |  |  |  |  | Y |
| 4 | *5430432M24Rik* | EG1969 |  |  |  |  |  |  | Y |
| 4 | *Gfra3* | EH2370 |  |  |  |  |  |  | Y |
| 4 | *AI467606* | EG1711 |  |  |  |  |  |  | Y |
| 4 | *Rab36* | EB1982 |  |  |  |  |  |  | Y |
| 4 | *1190002A17Rik* | EB2049 |  |  |  |  |  |  | Y |
| 4 | *Nktr* | EN175 |  |  |  |  |  |  | Y |
| 4 | *Rdbp* | EB2313 |  |  |  |  |  |  | Y |
| 4 | *Ubn1* | EG2173 |  |  |  |  |  |  | Y |
| 4 | *Usp29* | EG1532 |  |  |  |  |  |  | Y |
| 4 | *Ptpn2* | EH866 |  |  |  |  |  |  | Y |
| 4 | *Ankrd26* | EG1642 |  |  |  |  |  |  | Y |
| 5 | *Ctsz* | ES1059 |  |  |  |  |  |  | Y |
| 5 | *2410015M20Rik* | EB2076 |  |  |  |  |  |  | Y |
| 5 | *Tmem27* | EN527 |  |  |  |  |  |  | Y |
| 5 | *Tmed9* | EH1310 |  |  |  |  |  |  | Y |
| 5 | *Mid1ip1* | EG713 |  |  |  |  |  |  | Y |
| 5 | *Ccdc28b* | ES1884 |  |  |  |  |  |  | Y |
| 5 | *Prrg2* | EG727 |  |  |  |  |  |  | Y |
| 5 | *Plk2* | EH4027 |  |  |  |  |  |  | Y |
| 5 | *Nkiras2* | EG1759 |  |  |  |  |  |  | Y |
| 5 | *Efcbp2* | EG1460 |  |  |  |  |  |  | Y |
| 5 | *Banp* | EH2280 |  |  |  |  |  |  | Y |
| 5 | *Dennd2a* | EG1409 |  |  |  |  |  |  | Y |
| 5 | *Upp1* | EH2401 |  |  |  |  |  |  | Y |
| 5 | *Gpr120* | EN611 |  |  |  |  |  |  | Y |
| 5 | *1810037C20Rik* | ES216 |  |  |  |  |  |  | Y |
| 5 | *Trip11* | EH3128 |  |  |  |  |  |  | Y |
| 5 | *Fkbpl* | ES747 |  |  |  |  |  |  | Y |
| 5 | *Rnf6* | EB1710 |  |  |  |  |  |  | Y |
| 5 | *Pla2g2d* | MH1794 |  |  |  |  |  |  | Y |
| 5 | *Gpr119* | EH2809 |  |  |  |  |  |  | Y |
| 5 | *Rock1* | EB2039 |  |  |  |  |  |  | Y |
| 5 | *Zfp36* | EN543 |  |  |  |  |  |  | Y |
| 5 | *Sfxn2* | EN492 |  |  |  |  |  |  | Y |
| 5 | *Fosb* | EN1167 |  |  |  |  |  |  | Y |
| 5 | *Klc3* | EH1000 |  |  |  |  |  |  | Y |
| 5 | *Nudt16* | EH2374 |  |  |  |  |  |  | Y |
| 5 | *Plcxd3* | EG1331 |  |  |  |  |  |  | Y |
| 5 | *Ribc1* | EG913 |  |  |  |  |  |  | Y |
| 5 | *1700045I19Rik* | EB2021 |  |  |  |  |  |  | Y |
| 5 | *Tmem104* | EN1742 |  |  |  |  |  |  | Y |
| 5 | *Naglu* | ES1039 |  |  |  |  |  |  | Y |
| 5 | *Amigo2* | EB1761 |  |  |  |  |  |  | Y |
| 5 | *Rgl3* | EB201 |  |  |  |  |  |  | Y |
| 5 | *Micalcl* | EN1948 |  |  |  |  |  |  | Y |
| 5 | *Zfp560* | ES2431 |  |  |  |  |  |  | Y |
| 5 | *Pim2* | EN489 |  |  |  |  |  |  | Y |
| 5 | *Ttll3* | EG1930 |  |  |  |  |  |  | Y |
| 5 | *Atf6* | EN1201 |  |  |  |  |  |  | Y |
| 5 | *Guca2b* | ES2234 |  |  |  |  |  |  | Y |
| 5 | *Rom1* | EG1224 |  |  |  |  |  |  | Y |
| 5 | *Ifi35* | EB770 |  |  |  |  |  |  | Y |
| 5 | *2200002J24Rik* | ES1401 |  |  |  |  |  |  | Y |
| 5 | *Upb1* | EH991 |  |  |  |  |  |  | Y |
| 5 | *Rbks* | EB446 |  |  |  |  |  |  | Y |
| 5 | *Cnot4* | EH888 |  |  |  |  |  |  | Y |
| 5 | *1110032A03Rik* | EN281 |  |  |  |  |  |  | Y |
| 5 | *Pdia3* | EN82 |  |  |  |  |  |  | Y |
| 5 | *Neil1* | EB2563 |  |  |  |  |  |  | Y |
| 5 | *Mrpl27* | EG853 |  |  |  |  |  |  | Y |
| 5 | *Atpbd4* | EN1986 |  |  |  |  |  |  | Y |
| 5 | *S100pbp* | EB2366 |  |  |  |  |  |  | Y |
| 5 | *2410005O16Rik* | EH3785 |  |  |  |  |  |  | Y |
| 5 | *Kif16b* | EG2325 |  |  |  |  |  |  | Y |
| 5 | *Sgk2* | EN463 |  |  |  |  |  |  | Y |
| 5 | *Pla2g2c* | ES957 |  |  |  |  |  |  | Y |
| 5 | *AI842396* | EB2023 |  |  |  |  |  |  | Y |
| 5 | *Sec24a* | ES1650 |  |  |  |  |  |  | Y |
| 5 | *AI314976* | EN755 |  |  |  |  |  |  | Y |
| 5 | *Glis1* | EH2285 |  |  |  |  |  |  | Y |
| 5 | *Zfpl1* | ES681 |  |  |  |  |  |  | Y |
| 5 | *Med31* | ES1104 |  |  |  |  |  |  | Y |
| 5 | *Cln3* | EN78 |  |  |  |  |  |  | Y |
| 5 | *Centb2* | ES1759 |  |  |  |  |  |  | Y |
| 5 | *Cntfr* | EG673 |  |  |  |  |  |  | Y |
| 5 | *Pign* | EN1773 |  |  |  |  |  |  | Y |
| 5 | *Pccb* | MH1357 |  |  |  |  |  |  | Y |
| 5 | *D930048N14Rik* | EH3258 |  |  |  |  |  |  | Y |
| 5 | *Ms4a6c* | EG1808 |  |  |  |  |  |  | Y |
| 5 | *1700010I14Rik* | EN949 |  |  |  |  |  |  | Y |
| 5 | *Ap1s2* | EG552 |  |  |  |  |  |  | Y |
| 5 | *Dnaic2* | EG2358 |  |  |  |  |  |  | Y |
| 5 | *Ankrd55* | EN1159 |  |  |  |  |  |  | Y |
| 5 | *D030070L09Rik* | EN899 |  |  |  |  |  |  | Y |
| 6 | *BC023829* | EG1346 |  |  |  |  |  |  | Y |
| 6 | *Saal1* | EG2253 |  |  |  |  |  |  | Y |
| 6 | *Ildr1* | EB2412 |  |  |  |  |  |  | Y |
| 6 | *Igfals* | EH1043 |  |  |  |  |  |  | Y |
| 6 | *Tmc4* | EB1870 |  |  |  |  |  |  | Y |
| 8 | *Resp18* | EB2363 |  |  |  |  |  |  | Y |
| 8 | *Ush1g* | ES2028 |  |  |  |  |  |  | Y |
| 8 | *Nudt7* | EH1639 |  |  |  |  |  |  | Y |
| 8 | *Skap1* | EG1627 |  |  |  |  |  |  | Y |
| 9 | *Uba52* | EB2505 |  |  |  |  |  |  | Y |
| 9 | *Tbxa2r* | EN997 |  |  |  |  |  |  | Y |
| 10 | *Akr1e1* | EN709 |  |  |  |  |  |  | Y |
| 10 | *Mrc1* | EN236 |  |  |  |  |  |  | Y |
| 10 | *Fgfr1op2* | ES2012 |  |  |  |  |  |  | Y |
| 10 | *Aqp8* | EH3704 |  |  |  |  |  |  | Y |
| 10 | *Atp5e* | EN215 |  |  |  |  |  |  | Y |
| 10 | *3110062M04Rik* | EN729 |  |  |  |  |  |  | Y |
| 10 | *Stam2* | EB55 |  |  |  |  |  |  | Y |
| 10 | *Ube2b* | EN1626 |  |  |  |  |  |  | Y |
| 10 | *Icam4* | EH3583 |  |  |  |  |  |  | Y |
| 10 | *Fance* | ES2011 |  |  |  |  |  |  | Y |
| 10 | *Pdcd6ip* | ES2214 |  |  |  |  |  |  | Y |
| 10 | *1200009F10Rik* | EG775 |  |  |  |  |  |  | Y |
| 10 | *Pigw* | ES77 |  |  |  |  |  |  | Y |
| 10 | *Bik* | EG2025 |  |  |  |  |  |  | Y |
| 10 | *Fblim1* | EN1965 |  |  |  |  |  |  | Y |
| 10 | *Lst1* | EN1019 |  |  |  |  |  |  | Y |
| 10 | *Stard5* | ES264 |  |  |  |  |  |  | Y |
| 10 | *2810474O19Rik* | MH687 |  |  |  |  |  |  | Y |
| 10 | *Rabl3* | ES810 |  |  |  |  |  |  | Y |
| 10 | *Stk3* | MH1740 |  |  |  |  |  |  | Y |
| 10 | *Chek1* | EN1326 |  |  |  |  |  |  | Y |
| 10 | *Mbd4* | EB671 |  |  |  |  |  |  | Y |
| 10 | *1110038F14Rik* | EG817 |  |  |  |  |  |  | Y |
| 10 | *Trim35* | EN1684 |  |  |  |  |  |  | Y |
| 10 | *1810021J13Rik* | EN594 |  |  |  |  |  |  | Y |
| 10 | *Snapc5* | EG786 |  |  |  |  |  |  | Y |
| 10 | *A730011L01Rik* | EH3276 |  |  |  |  |  |  | Y |
| 10 | *Cxxc6* | ES1929 |  |  |  |  |  |  | Y |
| 10 | *Wscd2* | EN1730 |  |  |  |  |  |  | Y |
| 10 | *Myh6* | MH1768 |  |  |  |  |  |  | Y |
| 10 | *Acacb* | EB1782 |  |  |  |  |  |  | Y |
| 10 | *1810029B16Rik* | EN382 |  |  |  |  |  |  | Y |
| 10 | *P2ry6* | EG1809 |  |  |  |  |  |  | Y |
| 10 | *Mab21l2* | EH935 |  |  |  |  |  |  | Y |
| 10 | *Brwd1* | EG352 |  |  |  |  |  |  | Y |
| 10 | *Coq3* | EH526 |  |  |  |  |  |  | Y |
| 10 | *Gnpda1* | EN1372 |  |  |  |  |  |  | Y |
| 10 | *Terf2ip* | EH833 |  |  |  |  |  |  | Y |
| 10 | *5830433M19Rik* | EH3889 |  |  |  |  |  |  | Y |
| 10 | *Fsd2* | EH3637 |  |  |  |  |  |  | Y |
| 10 | *Afg3l2* | ES2333 |  |  |  |  |  |  | Y |
| 10 | *Ptpre* | EN242 |  |  |  |  |  |  | Y |
| 10 | *Rab2b* | EG599 |  |  |  |  |  |  | Y |
| 10 | *Yy1* | EB2473 |  |  |  |  |  |  | Y |
| 10 | *Dcp2* | EH3142 |  |  |  |  |  |  | Y |
| 10 | *Mcart1* | EB1466 |  |  |  |  |  |  | Y |
| 10 | *Anp32e* | EG1901 |  |  |  |  |  |  | Y |
| 10 | *Lrrc28* | ES113 |  |  |  |  |  |  | Y |
| 10 | *Sumo3* | MY297 |  |  |  |  |  |  | Y |
| 10 | *Nxt2* | EB1967 |  |  |  |  |  |  | Y |
| 10 | *Ogg1* | EB1815 |  |  |  |  |  |  | Y |
| 10 | *Arrdc4* | EG479 |  |  |  |  |  |  | Y |
| 10 | *BC030183* | EN533 |  |  |  |  |  |  | Y |
| 10 | *Ppp3r1* | EG1113 |  |  |  |  |  |  | Y |
| 10 | *2700081O15Rik* | ES1370 |  |  |  |  |  |  | Y |
| 11 | *Cpb1* | MH1601 |  |  |  |  |  |  | Y |
| 11 | *Sycn* | EH4032 |  |  |  |  |  |  | Y |
| 11 | *Anxa7* | EB777 |  |  |  |  |  |  | Y |
| 11 | *D830014E11Rik* | ES1767 |  |  |  |  |  |  | Y |
| 11 | *C76566* | EN1732 |  |  |  |  |  |  | Y |
| 11 | *Scly* | EB383 |  |  |  |  |  |  | Y |
| 11 | *Rbp2* | EB2000 |  |  |  |  |  |  | Y |
| 11 | *Ggt1* | EG1889 |  |  |  |  |  |  | Y |
| 11 | *Ccl11* | EG864 |  |  |  |  |  |  | Y |
| 11 | *Kcnc3* | EH3288 |  |  |  |  |  |  | Y |
| 11 | *Isg20* | EN371 |  |  |  |  |  |  | Y |
| 12 | *Pnlip* | EH2570 |  |  |  |  |  |  | Y |
| 12 | *Rnase1* | EH2505 |  |  |  |  |  |  | Y |
| 12 | *1810010M01Rik* | EB182 |  |  |  |  |  |  | Y |
| 12 | *Sparc* | EH1332 |  |  |  |  |  |  | Y |
| 12 | *Tm4sf1* | EN1690 |  |  |  |  |  |  | Y |
| 12 | *Fgl2* | EG1109 |  |  |  |  |  |  | Y |
| 12 | *Expi* | EG724 |  |  |  |  |  |  | Y |
| 12 | *Mmrn2* | EB2359 |  |  |  |  |  |  | Y |
| 12 | *Ushbp1* | EG1228 |  |  |  |  |  |  | Y |
| 12 | *Dmbt1* | EB2486 |  |  |  |  |  |  | Y |
| 12 | *Aqp12* | ES1382 |  |  |  |  |  |  | Y |
| 12 | *Crp* | EG1163 |  |  |  |  |  |  | Y |
| 12 | *Mall* | EB2245 |  |  |  |  |  |  | Y |
| 12 | *Cd14* | ES354 |  |  |  |  |  |  | Y |
| 12 | *Degs2* | ES1932 |  |  |  |  |  |  | Y |
| 12 | *Ehd4* | EN1640 |  |  |  |  |  |  | Y |
| 12 | *Pdgfd* | EG1690 |  |  |  |  |  |  | Y |
| 12 | *Sema3g* | EB2044 |  |  |  |  |  |  | Y |
| 12 | *Osmr* | EH1083 |  |  |  |  |  |  | Y |
| 12 | *Slco2b1* | ES1794 |  |  |  |  |  |  | Y |
| 13 | *Atp6ap2* | EG462 |  |  |  |  |  |  | Y |
| 13 | *G6pc2* | EN1339 |  |  |  |  |  |  | Y |
| 13 | *Slc33a1* | EG625 |  |  |  |  |  |  | Y |
| 13 | *Derl2* | EB754 |  |  |  |  |  |  | Y |
| 13 | *Pde4dip* | ES1084 |  |  |  |  |  |  | Y |
| 13 | *Gpr158* | EG2237 |  |  |  |  |  |  | Y |
| 13 | *Tmem157* | ES2361 |  |  |  |  |  |  | Y |
| 13 | *Adora3* | ES1378 |  |  |  |  |  |  | Y |
| 13 | *Apoc3* | EN160 |  |  |  |  |  |  | Y |
| 13 | *Snph* | ES2216 |  |  |  |  |  |  | Y |
| 13 | *Scnn1b* | EG87 |  |  |  |  |  |  | Y |
| 13 | *Sstr3* | EB2045 |  |  |  |  |  |  | Y |
| 13 | *Bcl6b* | EN1058 |  |  |  |  |  |  | Y |
| 13 | *Gpr98* | EB2554 |  |  |  |  |  |  | Y |
| 13 | *C030010B13Rik* | EG1669 |  |  |  |  |  |  | Y |
| 13 | *Slco1a6* | EN476 |  |  |  |  |  |  | Y |
| 13 | *Tspyl2* | EG581 |  |  |  |  |  |  | Y |
| 14 | *Rps9* | EN1276 |  |  |  |  |  |  | Y |
| 14 | *Rpl7* | EN94 |  |  |  |  |  |  | Y |
| 14 | *Dynll1* | EN264 |  |  |  |  |  |  | Y |
| 14 | *Sub1* | EN180 |  |  |  |  |  |  | Y |
| 14 | *D8Ertd738e* | EG1974 |  |  |  |  |  |  | Y |
| 14 | *Tbca* | EB2193 |  |  |  |  |  |  | Y |
| 1 | *Lgals2* | EH376 |  |  |  |  |  |  | Y |
| 1 | *Ihh* | EH613 |  |  |  |  |  |  | Y |
| 1 | *Rfc5* | EB2005 |  |  |  |  |  |  | Y |
| 3 | *Camp* | EH569 |  |  |  |  |  |  | Y |
| 4 | *Samd8* | ES1646 |  |  |  |  |  |  | Y |
| 5 | *4930488E11Rik* | EN1806 |  |  |  |  |  |  | Y |
| 5 | *Etfa* | ES457 |  |  |  |  |  |  | Y |
| 5 | *Mia1* | EN1751 |  |  |  |  |  |  | Y |
| 5 | *Scpep1* | EB1141 |  |  |  |  |  |  | Y |
| 5 | *Tpst2* | EH836 |  |  |  |  |  |  | Y |
| 5 | *Cbfa2t3h* | EN1686 |  |  |  |  |  |  | Y |
| 5 | *Ms4a7* | ES2175 |  |  |  |  |  |  | Y |
| 5 | *Edn3* | EG1459 |  |  |  |  |  |  | Y |
| 5 | *Rab11b* | ES1045 |  |  |  |  |  |  | Y |
| 5 | *Bbx* | ES134 |  |  |  |  |  |  | Y |
| 5 | *Phactr4* | EN1181 |  |  |  |  |  |  | Y |
| 5 | *Smarcb1* | EB761 |  |  |  |  |  |  | Y |
| 5 | *Gnas* | EH3795 |  |  |  |  |  |  | Y |
| 5 | *Nat5* | EH2567 |  |  |  |  |  |  | Y |
| 5 | *4930455F23Rik* | EG619 |  |  |  |  |  |  | Y |
| 5 | *Dok2* | EB50 |  |  |  |  |  |  | Y |
| 5 | *Nvl* | MH1246 |  |  |  |  |  |  | Y |
| 5 | *Tex261* | ES547 |  |  |  |  |  |  | Y |
| 5 | *Guca2a* | EH167 |  |  |  |  |  |  | Y |
| 5 | *Prpf39* | EH2750 |  |  |  |  |  |  | Y |
| 6 | *Dnajc18* | EN1913 |  |  |  |  |  |  | Y |
| 6 | *Sdpr* | EG288 |  |  |  |  |  |  | Y |
| 6 | *Arfgap1* | EH841 |  |  |  |  |  |  | Y |
| 6 | *Sec24c* | ES424 |  |  |  |  |  |  | Y |
| 10 | *H19* | EN370 |  |  |  |  |  |  | Y |
| 10 | *Phf12* | EB1340 |  |  |  |  |  |  | Y |
| 10 | *Cd47* | EN867 |  |  |  |  |  |  | Y |
| 10 | *5730494M16Rik* | ES912 |  |  |  |  |  |  | Y |
| 10 | *Limk1* | MH344 |  |  |  |  |  |  | Y |
| 11 | *Anxa5* | ES561 |  |  |  |  |  |  | Y |
| 11 | *Gtl2* | EH2054 |  |  |  |  |  |  | Y |
| 12 | *Plvap* | EG1184 |  |  |  |  |  |  | Y |
| 12 | *Lims2* | EB2244 |  |  |  |  |  |  | Y |
| 12 | *1810015C04Rik* | EG420 |  |  |  |  |  |  | Y |
| 12 | *Vnn1* | EN478 |  |  |  |  |  |  | Y |
| 12 | *2210023G05Rik* | EH1950 |  |  |  |  |  |  | Y |
| 14 | *Prdx2* | ES1286 |  |  |  |  |  |  | Y |
| 14 | *Ngfrap1* | EG1735 |  |  |  |  |  |  | Y |
| 14 | *Pabpc1* | EG1112 |  |  |  |  |  |  | Y |
| 1 | *Bcl2* | EH2436 |  |  |  |  |  |  | Y |
| 2 | *Tpp2* | EB1304 |  |  |  |  |  |  | Y |
| 3 | *Kcnj8* | ES600 |  |  |  |  |  |  | Y |
| 3 | *Tac2* | EB414 |  |  |  |  |  |  | Y |
| 3 | *Mrps23* | EH415 |  |  |  |  |  |  | Y |
| 3 | *Ptger3* | EN923 |  |  |  |  |  |  | Y |
| 4 | *Nisch* | EG2012 |  |  |  |  |  |  | Y |
| 4 | *Rab14* | EB1138 |  |  |  |  |  |  | Y |
| 4 | *Hist1h1e* | EN1016 |  |  |  |  |  |  | Y |
| 4 | *Gtpbp10* | EB1894 |  |  |  |  |  |  | Y |
| 5 | *Med28* | EH2340 |  |  |  |  |  |  | Y |
| 5 | *Dnaja1* | EB594 |  |  |  |  |  |  | Y |
| 5 | *Trrap* | EH4036 |  |  |  |  |  |  | Y |
| 5 | *Wbp5* | EH659 |  |  |  |  |  |  | Y |
| 5 | *Glul* | ES94 |  |  |  |  |  |  | Y |
| 5 | *Rbm5* | EB1497 |  |  |  |  |  |  | Y |
| 5 | *Pxmp4* | EH1674 |  |  |  |  |  |  | Y |
| 5 | *Spint1* | EN606 |  |  |  |  |  |  | Y |
| 5 | *Atpaf2* | EB1094 |  |  |  |  |  |  | Y |
| 5 | *Mrpl19* | EH1044 |  |  |  |  |  |  | Y |
| 5 | *Rbed1* | EH1585 |  |  |  |  |  |  | Y |
| 5 | *Cdc23* | EB1633 |  |  |  |  |  |  | Y |
| 5 | *Ccdc59* | EN414 |  |  |  |  |  |  | Y |
| 5 | *Txlna* | EN1066 |  |  |  |  |  |  | Y |
| 5 | *Aqp7* | EB1388 |  |  |  |  |  |  | Y |
| 5 | *Ash1l* | EB1885 |  |  |  |  |  |  | Y |
| 5 | *Mrps12* | EB10 |  |  |  |  |  |  | Y |
| 5 | *Ppp2r3a* | EH3751 |  |  |  |  |  |  | Y |
| 5 | *Efcab3* | EN1816 |  |  |  |  |  |  | Y |
| 6 | *Mtf1* | EH860 |  |  |  |  |  |  | Y |
| 6 | *Wdr32* | EH1989 |  |  |  |  |  |  | Y |
| 10 | *Acat1* | EG995 |  |  |  |  |  |  | Y |
| 10 | *Kbtbd4* | EH413 |  |  |  |  |  |  | Y |
| 10 | *Eif4a2* | EB1488 |  |  |  |  |  |  | Y |
| 10 | *Rcbtb2* | EN1130 |  |  |  |  |  |  | Y |
| 10 | *D19Wsu162e* | EH2700 |  |  |  |  |  |  | Y |
| 11 | *Isoc1* | EN626 |  |  |  |  |  |  | Y |
| 11 | *BC029169* | EN385 |  |  |  |  |  |  | Y |
| 12 | *Ccdc21* | EN758 |  |  |  |  |  |  | Y |
| 12 | *Il17re* | EN1933 |  |  |  |  |  |  | Y |
| 12 | *Cxcl16* | EH3763 |  |  |  |  |  |  | Y |
| 13 | *Zc3h3* | ES1182 |  |  |  |  |  |  | Y |
| 14 | *Pfn1* | ES636 |  |  |  |  |  |  | Y |
| 14 | *Rps5* | EB1646 |  |  |  |  |  |  | Y |
| 14 | *Ddx6* | EG216 |  |  |  |  |  |  | Y |
| 1 | *Myog* | EH1977 |  |  |  |  |  |  | Y |
| ^o^ The accession number of the gene in the GenePaint database [62].  ^ W indicates weak staining, M indicates moderate staining, and S indicates strong staining  ^#^ Not detected indicates that the probe for the gene produced staining in the embryo, suggesting the probe was functional, but no staining was seen in the pancreas  ^Δ^ Undetermined suggests either that the probe produced no noticeable stain on any section, or sections containing pancreas were absent, or the data was not interpretable | | | | | | | | | |
