# Supplementary material for: Identification of transcripts with enriched expression in the developing and adult pancreas
Source: Genome Biol. 2008 Jun 14;9(6):R99. doi: 10.1186/gb-2008-9-6-r99 (PMC2481431; doi:10.1186/gb-2008-9-6-r99)
Supplement: Additional data file 5 — Genes and primer sequences used in qRT-PCR validation studies. [file gb-2008-9-6-r99-S5.doc]

| **Table S3:** Primer sequences | | |
| --- | --- | --- |
| Gene Symbol | L-Primer Sequence | R-Primer Sequence |
| Primers for qRT-PCR | | |
| *Onecut2* | CACCACGCCATGAGTATGTC | GGCGTCAGCGTAGTGTAGGT |
| *Sfrp5* | TGGACAACGACCTCTGCAT | TGTGCTCCATCTCACACTGG |
| *Crabp2* | GCCGAGAACTGACCAATGAT | GGAAGTCGTCTCAGGCAGTT |
| *Fusip1* | CACGTCTCTGTTCGTCAGGA | GGACGCCGAGTGTAGAAATC |
| *Rbp1* | GTGGATCGAGGGTGATGAAC | AGGTTATCTCCTCGGGCTGT |
| *Fh1* | GCTGCAGTGTCTGGGGAAG | TCGGTTGGAACCTTCAATTC |
| *Sfrp1* | TCAGAGGCCATCATTGAACA | CCCAGCTTCAAGGGTTTCTT |
| *Ambp* | CCTTCTGCAGGAGTTCAAGG | GATCCCCAGGGACACATTC |
| *Habp2* | CTCTGGCTGGGGTGTTACAG | GGGAGTTGCACAAAGGGTTA |
| *F11r* | GCGTCGGGATTGTAACTGTAA | AGTGTACACCGAACCCTTGC |
| *Cryba2* | TACTGAGCGACTGTGCCAAC | ATGAACTGCTGTCCCTGGAA |
| *Slc38a5* | CAAATCTGAACTCCCCCTTG | CCAGGGGCAAGATGATTAAC |
| *Gast* | CCCAGGGTCCTCAACACTT | ATCCATCCGTAGGCCTCTTC |
| *AI987662* | CATGAAGATGCCCTGAGTGA | TGCCCTGTTACACCATCAGA |
| *Rbp4* | GGCAGTACAGATGGATTGAACA | GATGAAGACCGGATGAAAGC |
| *Cdkn1a* | GTCTGAGCGGCCTGAAGAT | TCTGCGCTTGGAGTGATAGA |
| *Tekt2* | GACGCTGGACAAGTGTCTGA | GTTCTTGGCCTGCAGGTTT |
| *Tle6* | AGACCCAGAAAGCCACTTGA | GTCCTGCTGTTGGAGGACAG |
| *Irx2* | GCATTCACTGGAGTCCCACT | ACAACTGCACAGCCCTCACT |
| *St14* | CGCGGGACTCAAGTACAACT | TCGCTTCTCCACTTTCTTGG |
| *E430002G05Rik* | CAAGAACTTCGATGGCTTCC | AAGACCCAGTGGTGTCAAGG |
| *Abcc8* | GCTCAGACAGCACACTCTGG | CTCCCCTCCTTCTGTGATGA |
| *Insrr* | GTTCGAGAACCACCTTCAGC | CTCACTGTGGCAGCAATCAC |
| *Mlxipl* | CCCCTCACTCAGGGAATACA | CAGAGCTCAGAAAGGGGTTG |
| *Myt3* | AAGCCCAAGCTGCATACAAG | TGCGGTGAGATGCATAGTTG |
| *Cdkn1c* | CTGAAGGACCAGCCTCTCTC | GTTCTCCTGCGCAGTTCTCT |
| *Nkx2-3* | ACAGTTCTCCGAGACGCTTG | CCGCTTCCAGAGACTTCTTC |
| *Rbpjl* | AGCAGTCGCTGAAAAACACA | AGAGAGGTAACGCGTGGACA |
| *P2rx1* | CCAGTTGGTGGTTCTGGTCT | GCTGATAAGGCCACTTGAGG |
| *Nr2f6* | GGACAAGTCCAGTGGAAAGC | TCTGACAGTCACGGTTGGAC |
| *Hhex* | ACTACACGCACGCCCTACTC | GCCTTTCCTTTTGTGCAGAG |
| *Clu* | CAGCTGGCTAACCTCACACA | AACAGCTTCACCACCACCTC |
| *Dusp1* | TGTGCCTGACAGTGCAGAAT | CTTCCGAGAAGCGTGATAGG |
| *Sytl4* | AGTCCCTCCTTCAGCAGACA | CTTTGGCACTTCAAACAGCA |
| *Rgs11* | ATATACAAGGGCCTGCTGGA | CTTCCGCAAGAATGGAAATG |
| *BC038479* | GACCAGGGCCCTACATCTG | GTGAAGCCGTGGTAGGTTGT |
| *Ins2* | from Applied Biosystems |  |
| *Mafa* | CACCACCACGGAGGCTCT | AGCTGGTCGTCGGAGAAG |
| *Pdx1* | from Applied Biosystems |  |
| *Neurog3* | from Applied Biosystems |  |
| *Ptf1a* | from Applied Biosystems |  |
| *Nkx2-2* | from Applied Biosystems |  |
| *Arx* | TCTCTGAGCCCAAGGAAAA | TGGTCTTGAGTGGTGCTGAG |
| *Amy* | from Applied Biosystems |  |
| Primers for ChIP-qPCR (Target-Binding factor) | | |
| *Foxa2-Foxa2BS* | TGAAGTCATCCCACAAGGCCCATT | CCAAGGTGCCCAAAGCATTTCGTA |
| *Foxa2-Pdx1BS* | GCCCAAAGCATTTCGTAACT | CCACAAGGCCCATTATTGAT |
| *Ins1-Pdx1BS* | TCAGCCAAAGATGAAGAAGGTCTC | TCCAAACACTTGCCTGGTGC |
| *Myt1-Foxa2BS* | TTTGGAGCACCTCTCACCTT | GCTACCCATGAACCCTTGG |
| *Myt1-Pdx1BS* | GTTTTGTTTGCTCCATCTGC | AGCCGAGATGGTCAGCATTA |
| *Myt3-Foxa2BS* | AAAGTGTGGCTGCCTTTGTC | AGGAGCGCTGTCACTTGG |
| *Neurod1-Foxa2BS* | TTTGCAATGAATGCATCCTAC | CCAGATGTACCCCATTCCAG |
| *Neurod1-Pdx1BS* | ACTAATGCCCCCTCTTAGGC | GCCACCCCAGTAGTCTTGTC |
| *Nkx22-Foxa2BS* | CCAGCTGAACAATCACTGGA | TTAGAGGGCCACTTGTCCTG |
| *Nkx22-Pdx1BS* | ACGTTCCCCATTCCTCTTTC | AGGAACACGTTTCAAACCAGA |
| *Nkx6.1 Pdx1BS* | AAGCTGATTTCATCCCCAGA | CATCTTTCCGTCTCCTGCTC |
| *Nkx61-Foxa2BS* | TAAACACCGCCTCCAATAGC | TCAGCCAATCAAAAGGTGTG |
| *Pdx1 Pdx1BS* | CATTCCCTTATCACATGCTCA | TGTGACCAAACAGCTTCCTT |
| *Pdx1-Foxa2BS* | TGCCCTTTACTCAGGAGTGG | GCCATCCAGATGACTAAGAGTG |
