# Supplementary material for: Identification of transcripts with enriched expression in the developing and adult pancreas
Source: Genome Biol. 2008 Jun 14;9(6):R99. doi: 10.1186/gb-2008-9-6-r99 (PMC2481431; doi:10.1186/gb-2008-9-6-r99)
Supplement: Additional data file 6 — Binding information for identified transcription factors. [file gb-2008-9-6-r99-S6.doc]

| **Table S4:** Binding Motifs for Identified Transcription Factors | | |
| --- | --- | --- |
| Gene symbol | Reported Binding Motif | Sequenced Used to Search CisRED |
| *Arid3a* | A/GATT/AAA[66] | RATWAA |
| *Arx* | na |  |
| *Asb4* | na |  |
| *Bcl6b* | na |  |
| *Elf3* | GAGGGAAAC / CAGGAAAC[67] |  |
| *Fev* | RRMAGGAARTR [68] | RRMAGGAARTR |
| *Fos* | Transfac | AP1 |
| *Foxa1* | Transfac | CisRED |
| *Foxa2* | Transfac | TRTTTRY |
| *Foxa3* | Transfac | CisRED |
| *Hes6* | Transfac | CisRED |
| *Hnf1a* | Transfac | CisRED |
| *Hnf4a* | Transfac | CisRED |
| *Hoxb5* | T/AA/TATTA [69] | WWATTA |
| *Hoxb6* | T/AA/TATTA [69] | WWATTA |
| *Ipf1* | Transfac | CisRED |
| *Isl1* | CTAATG [70] | CTAATG |
| *Mafa* | TGC T/A G A/C C/T TCAGCC [48] | TGCWGMYTCAGCC |
| *Mafb* | Transfac | CisRED |
| *Meis1* | TGACAG/A [71] | TGACAR |
| *Meox1* | TAATTA [72] | WWATTA |
| *Mlxipl* | CACGTG [73] | CACGTG |
| *Myt1* | AAAGTTT[74] | AAASTTT |
| *Myt3* | AAAGTTT[75] | AAASTTT |
| *Neurod1* | CANNTG [76] | CANNTG |
| *Neurog3* | CANNTG | CANNTG |
| *Nkx2-2* | Transfac | CisRED |
| *Nkx6-1* | T/A T/C TTAAT T/G G/T | WYTTAATKK |
| *Nkx6-2* | Transfac | CisRED |
| *Nr1d1* | WAWNTRGGTCA [77] | WAWNTRGGTCA |
| *Onecut1* | DHWATTGAYTWWD | DHWATTGAYTWWD |
| *Onecut2* | AAATCAATW [78] | AAATCAATW |
| *Pax4* | Transfac | CisRED |
| *Pax6* | Transfac | CisRED |
| *Pou3f4* | ATTATTTA [79] | ATTATTTA |
| *Preb* | na |  |
| *Rnf6* | GGGGC Nn GGGGC [80] |  |
| *Rorc* | na |  |
| *Son* | GA(G/T)AN(C/G)(A/G)CC [81] | GAKANSRCC |
| *Sox9* | Transfac | CisRED |
| *Tcf12* | CANNTG | CANNTG |
